# Eating Disorders During Gestation: Implications for Mother's Health, Fetal Outcomes, and Epigenetic Changes

**DOI:** 10.3389/fped.2020.00587

**Published:** 2020-09-17

**Authors:** Giorgia Sebastiani, Vicente Andreu-Fernández, Ana Herranz Barbero, Victoria Aldecoa-Bilbao, Xavier Miracle, Eva Meler Barrabes, Arantxa Balada Ibañez, Marta Astals-Vizcaino, Silvia Ferrero-Martínez, María Dolores Gómez-Roig, Oscar García-Algar

**Affiliations:** ^1^Neonatal Unit, Hospital Clinic-Maternitat, Institut Clinic de Ginecologia, Obstetricia i Neonatologia (ICGON), Barcelona Center for Maternal Fetal and Neonatal Medicine (BCNatal), Barcelona, Spain; ^2^Grup de Recerca Infancia i Entorn (GRIE), Institut d'investigacions Biomèdiques August Pi i Sunyer (IDIBAPS), Barcelona, Spain; ^3^Valencian International University (VIU), Valencia, Spain; ^4^Fetal i+D Fetal Medicine Research Center, BCNatal-Barcelona Center for Maternal-Fetal and Neonatal Medicine (Hospital Clínic and Hospital Sant Joan de Déu), IDIBAPS, University of Barcelona, Barcelona, Spain; ^5^Hospital Sant Joan de Déu, Barcelona Center for Maternal Fetal and Neonatal Medicine (BCNatal), Barcelona, Spain

**Keywords:** eating disorders, anorexia nervosa, bulimia nervosa, binge eating disorders, pregnancy, fetal outcomes, epigenetics, maternal psychopathology

## Abstract

**Introduction:** Eating disorders (EDs) have increased globally in women of childbearing age, related to the concern for body shape promoted in industrialized countries. Pregnancy may exacerbate a previous ED or conversely may be a chance for improving eating patterns due to the mother's concern for the unborn baby. EDs may impact pregnancy evolution and increase the risk of adverse outcomes such as miscarriage, preterm delivery, poor fetal growth, or malformations, but the knowledge on this topic is limited.

**Methods:** We performed a systematic review of studies on humans in order to clarify the mechanisms underpinning the adverse pregnancy outcomes in patients with EDs.

**Results:** Although unfavorable fetal development could be multifactorial, maternal malnutrition, altered hormonal pathways, low pre-pregnancy body mass index, and poor gestational weight gain, combined with maternal psychopathology and stress, may impair the evolution of pregnancy. Environmental factors such as malnutrition or substance of abuse may also induce epigenetic changes in the fetal epigenome, which mark lifelong health concerns in offspring.

**Conclusions:** The precocious detection of dysfunctional eating behaviors in the pre-pregnancy period and an early multidisciplinary approach comprised of nutritional support, psychotherapeutic techniques, and the use of psychotropics if necessary, would prevent lifelong morbidity for both mother and fetus. Further prospective studies with large sample sizes are needed in order to design a structured intervention during every stage of pregnancy and in the postpartum period.

## Introduction

Pregnancy is an exceptional condition that implicates intense psychological and biological transformations, which may change the perception of body shape, as well as influence modifications in eating patterns. Pregnancy therefore represents a period of increased weakness that triggers or aggravates symptoms of problematic eating behaviors (1).

According to the Diagnostic and Statistical Manual of Mental Disorders Fifth edition (DSM-V), Eating Disorders (EDs) are “disturbances in eating behavior that result in altered consumption of food and that significantly impair physical health and psychosocial functioning” (2). Anorexia Nervosa (AN) is characterized by a restriction of energy intake, leading to significant weight loss, intense anxiety around gaining weight, and a distorted perception of body weight and shape, associated with perfectionism and overcontrol. Bulimia Nervosa (BN) is associated with recurrent episodes of binge eating (eating a great amount of food in a discrete period of time with a sense of lack of control over eating and impulsivity), accompanied by inappropriate compensatory habits such as self-induced vomiting, abuse of laxatives or diuretics, and/or excessive exercise. Binge-eating disorder (BED) is defined by recurrent episodes of binge eating more rapidly than normal, which are followed by feeling uncomfortable, disgusted, depressed, or guilty as a result, however it is not associated with compensatory behaviors to prevent weight gain. Other Specified Feeding of Eating Disorders (OSFED) or Eating Disorders Not Otherwise Specified (EDNOS) refer to heterogeneous symptoms of feeding or ED resulting in a clinically significant problem that does not meet the full criteria of others disorders (2). Patients with chronic EDs often change between types of EDs (most commonly between AN and BN).

EDs are common in women of reproductive age. The prevalence of AN is an estimated 7 per 1,000 in the UK, with more incidence in adolescent girls and young women. BN is more frequent and affects older age groups, with a prevalence of 0.5–1% in women of reproductive age (3). Atypical EDs are probably more common but the literature about their prevalence is scarce. According to the American Association of EDs, at least 0.9% of American women suffer from AN, 1.5% from BN, and 2.8% from BED in their lifetime (4). In Europe, AN is reported at 1–4%, BN at 1–2%, BED at 1–4%, and subthreshold eating disorders at 2–3% of women (5). Some recent epidemiological studies estimate that the percentage of EDs has increased over the last years to ~5–10% of women of childbearing age (6).

The prevalence of EDs has been estimated at up to 7.5% in pregnant women (7). The incidence of EDs in the Norwegian Mother and Child Cohort was about 5%, where they found high incidence of BED in particular (8). In Italy the prevalence of AN, BN, and purging disorders during gestation was valued at up to 5.5% (9). A study conducted on pregnant Brazil population showed a prevalence of 17.3% of binge eating patterns associated with anxiety and mood liability (10).

Pregnancy and the postpartum period are related to variations in eating patterns such as overeating, strong rejection of specific foods or drinks, changes in taste perception, and women experience major body changes unseen since adolescence, which may trigger an ED in otherwise healthy pregnant patients (11, 12). Our culture which promotes slimness, weight concern and dissatisfaction with gaining weight, might lead to more dysfunctional eating attitudes (13, 14), because pregnant women may feel stressed and anxious over their changing body shape (15–17). In addition, the adaptive neuroendocrine modifications during gestation may alter brain function, impairing metabolism, appetite regulation, and mood (18).

Nevertheless, for most women ED symptoms tend to reduce during pregnancy because of the concern for the fetus, however this transient improvement of symptoms may revert in the postpartum period (19). A retrospective study reported that the prevalence of EDs was 11.5% from 3 to 7 months post-partum, with the most incidence in younger women (20), and a longitudinal study found that 12.8% of postpartum mothers suffered from a clinical eating disorder (21). This underlines that the postnatal period represents a vulnerable time for the exacerbation of disordered eating, probably due to anxiety about the postpartum body shape and to the frustration and stress of taking care of demanding infants, especially if there is not enough marital support (22). Furthermore, a longitudinal case-control study underlined that women with a recent ED had a higher rate of concern about weight gain and more loss of control over eating during pregnancy, and women with a past ED presented more compensatory behaviors than controls (23). In cross-sectional data in a Norwegian pregnant population, the incidence of BED during gestation was linked to social problems, symptoms of anxiety and depression, low life satisfaction, history of sexual and physical abuse, and smoking. Apprehension about pregnancy-related weight gain was the variable most strongly related to the onset of BED (24). Dysfunctional eating patterns may also reflect stress-response mechanisms related to the activation of Hypothalamic-pituitary-adrenal (HPA) axis and the corticotropin-releasing hormone (CRH) secretion which modulate food intake. Stress both stimulates the proopiomelanocortin (POMC) neurons eliciting anorexic signals and induces suppression of NPY secretion, declining its central orexigenic and anxiolytic actions (25).

Additionally, there are cultural influences that may trigger an ED during pregnancy. For example, food restriction during pregnancy is common in Chinese women, owing to the belief that this may protect the child and avoid complications such as miscarriage, stillbirth, death of the mother, and imperfections in newborns. For this reason, traditional women avoid cold foods or wet-hot foods, which are considered bad for the baby. These restrictions are often associated with symptoms of depression (26). Recent evidence showed that maternal disordered eating behaviors impact the course of pregnancy and fetal development. Updated reviews of the literature highlighted that the major obstetric and gynecologic complications were infertility, high rate of miscarriage, poor nutrition during pregnancy, hyperemesis gravidarum, cesarean section, preterm delivery, and postpartum depression (27–30). The most described detrimental effects on fetal development were fetal growth delay, small for gestational age babies and small head circumference, low Apgar score and an increased risk of perinatal mortality (27–30).

Despite the risk of EDs for both mother and fetus, the recognition of EDs during pregnancy is considered a challenge. ED is a multifactorial pathology, and it may be linked to predisposing factors including genetics, familiar and social dysfunction, mental illness such as Obsessive-Compulsive Personality Disorder (in AN) and Borderline Personality Disorder (in BN), or psychiatric disorders such as anxiety, depression, and substance abuse (31). There are not specific biochemical markers or precise instruments for the diagnosis of EDs in pregnant population, but standardized screening method, and questionnaires are available, which may differentiate normative dieting and eating concern from more dysfunctional behaviors (32, 33) (see Supplementary Material). Moreover, it is difficult to recognize the onset of an ED, because women often keep their condition secret (34). Stigmatizing attitudes may contribute to feelings of shame and guilt that lead them to hide their disorder and avoid help (35). On the other hand, clinical symptoms during pregnancy may be masked due to a reduction in clinical features or the presence of pregnancy sickness and hyperemesis gravidarum (36). Nonetheless, it is imperative to reach an early diagnosis in order to prevent long-lasting effects on women of reproductive age and impaired fetal outcomes (37). Clinicians should pay attention to warning signs such as lack of weight gain in consecutive visits during the second trimester, hyperemesis gravidarum that persists more than 20 weeks, or previous history of ED, depression, or dieting (38).

The purpose of this systematic review was to provide an update on the existing studies in humans focused on the effects of EDs during pregnancy on maternal and fetal outcomes. Specifically, our aim was to increase our knowledge on the topic of EDs during pregnancy, in order to highlight the importance of early prevention of these disturbances. We also focused on the effect of these dietary patterns have on fetal development and maternal psychopathology, to find a target therapy that could avoid fetal complications.

## Methods

The manuscripts included in this systematic review mainly concern EDs such as AN, BN, BED, and ENDOS. We did not assess studies about other types of EDs. Studies were screened based on PRISMA methodology, by searching titles and abstracts in the following electronic databases: SCOPUS, MEDLINE//PubMed, and Cochrane. The research was based on the combination of the following descriptors: eating disorders, anorexia nervosa, bulimia nervosa, binge eating disorders, pregnancy outcomes, fetal development, small for gestational age, epigenetics, preterm delivery, malformations, food intake, eating patterns, fertility, gestational weight gain, postpartum depression, maternal psychopathology, and breastfeeding/human milk. Boolean terms AND/OR/XOR were combined in the search.

Inclusion criteria were: the period covered from January 1 of 2000 to May 31, 2020, the presence of selected descriptors in the title of the papers or as keywords, only articles written in English language and only studies performed in humans, excluding books and documents as well as all references which results were based on animal models. Original manuscripts were selected by screening titles and abstracts, creating a reference list of relevant papers for the topics explored in this review. Of note, pre-conception period was also included. Two investigators (A.-F.V. and S.G.) conducted each stage of the studies selection, deleted duplicate inputs and evaluated the quality of the studies. All data were extracted by one investigator (S.G.) and cross-checked by a second investigator (A.-F.V.). In case of discrepancies in the selected studies, we opted for reconciliation through team discussion. The following variables were also explored: study characteristics including study design (case–control, review, longitudinal cohort, and cross-sectional), sample size, country, characteristics of participant population (number of cases, diagnostic criteria for EDs, hospitalized patients, type of EDs) and results (including strengths, limitations, conclusions, and possible biases). The quality of controlled studies (randomized, non-randomized, before-after) was critically appraised using the Cochrane Collaboration's Risk of Bias Tool (39).

The initial search identified a total of 249 papers once the inclusion and exclusion criteria were applied. After reviewing the abstract 126 studies were not selected because they were out of the scope of this review (eating disorders, pregnancy and fetal outcomes) and we also discarded 11 duplicate studies. Thirty records were selected through other sources by their relevance in the field. This resulted in 142 published studies. Additionally, for epigenetics section, the search was “(eating disorders or anorexia nervosa or bulimia nervosa or binge eating disorders) and (pregnancy or fetal outcome or preterm) and epigenetics” obtaining 25 results. Twenty articles were selected and 5 additional papers were included from other sources by their relevance in this issue. In the case of breastfeeding section, the terms used were “(eating disorders or anorexia nervosa or bulimia nervosa or binge eating disorders) and (pregnancy or fetal outcome or preterm) and breastfeeding” showing 42 results. Thirty studies were finally selected. Both sections were performed following the same inclusion and exclusion criteria. Finally, 197 studies were selected to perform this review.

We expected an important bias due to the heterogeneous results observed in the literature selected by the different populations compared, the distinct health condition of them, the lack of randomized trials in pregnant women with EDs the use of questionnaires to detect nutritional deficits and psychological alterations as well as the small sample size observed in some studies. Otherwise Table S1 included the American Dietetic Association Guidelines for pregnancy and the International Guidelines for the management of EDs during pregnancy to contextualize the nutritional requirements during this period. Moreover, scientific papers related to the nutritional and hormonal status of pregnant women were also included to evaluate possible detrimental effects on fetal development.

## Review

### Periconceptional Period in Women With EDs

Women with EDs are reported to have menstrual irregularity, problems with sexuality linked to a conflicted relationship with their body, decreased libido, and high sexual anxiety (40, 41). This can lead to insufficient use of contraception methods with a consequent unplanned pregnancy. In a prospective pregnancy cohort study, 50% of women with AN reported unplanned pregnancy when compared to a control group without EDs (42). An unplanned pregnancy may add risk to fetal development due to poor prenatal care, such as drinking alcohol, failing in balanced nutrition, or delaying the intake of prenatal vitamins. Moreover, unintentional pregnancy may exacerbate contrasting feelings in women, such as excitement yet depression and anxiety, when discovering their pregnant status.

There is a critical level of accumulated energy or fat mass that is essential for maintaining regular menstruation. EDs may affect fertility and menstruation because malnutrition and weight loss may alter hormonal status. Reduced levels of luteinizing hormone (LH) and follicle-stimulating hormone (FSH) suggest hypothalamic and pituitary dysfunction, which often lead to anovulation and amenorrhea. Sex hormones interact with neurotransmitters to achieve central control of appetite and energy balance, so in EDs there is a deregulation of appetite mechanisms (43). Women with AN may experience a drop in levels of leptin, a hormone that inhibits appetite and it is linked to energy homeostasis. This fall in leptin is associated with low caloric intake and with low fat mass, resulting in the reduction of the pulsatile release of gonadotropin-releasing-hormone (GnRH). All these hormonal pathways provoke dysfunction in ovulation, menstruation, and bone growth (44). Moreover, it has been reported that women with BN may be affected by hyperandrogenism and polycystic ovary syndrome (PCOS), a common cause of infertility, characterized by higher levels of circulating androgens. Testosterone is known to stimulate appetite and has been linked to altered impulse control and depression, so patients with PCOS exhibit a deregulation of appetite that may exacerbate bulimic symptoms (45). In addition, elevated ghrelin levels observed in patients with EDs may produce continuing inhibition of the hypothalamic-pituitary-ovarian axis and amenorrhea in spite of normal body fat and leptin levels. Low insulin status is also linked to amenorrhea, related to hypothalamic sensors of insulin levels. In patients with EDs, the activation of the HPA axis starts as a result of a stress reaction, leading to increased levels of CRH and cortisol, and failure of the circadian rhythm of cortisol. Through metabolic pathways, these hormones interact with the GnRH directly or through neuropeptide mediators (neuropeptide Y or kisspeptin). The final result of these metabolic signals in patients with EDs, is the inhibition of the normal pulsatile GnRH, low levels of LH, FSH, estradiol and small ovaries, the main causes of infertility (46).

There is little data about the prevalence of EDs in women affected by fertility problems. The prevalence of ovulatory disturbances is considerably higher in females with severe EDs (47). A recent longitudinal study conducted on women who started infertility treatment, found that the prevalence of EDs was 20.7% and the most prevalent EDs were AN and BED (48). In the longitudinal Avon study cohort of Parents and Children (ALSPAC) (49), the EDs groups with AN or AN+BN participants, took no longer than 12 months to conceive, but they delayed more than the general population (>6 months), and were more likely to conceive the current pregnancy with fertility treatment. This study underlines that women with AN, or AN+BN experienced conception problems without meeting the criteria for infertility (defined as inability to conceive for more than 12 months). This group included women with the lowest preconception BMI, which may explain the difficulty in conceiving. The authors concluded that fertility was affected in the EDs group but not considerably impaired. The limitation of this study is that the classification of EDs was made on the basis of self-reporting. A recent longitudinal population-based cohort study performed in the Netherlands showed that EDs were associated with increased odds of receiving fertility treatment and twin births, regardless of pre-pregnancy BMI (50). Another study showed that female patients with EDs recruited at Helsinki University Central Hospital had impaired reproductive health with the lowest pregnancy rate in patients with AN and a high rate of miscarriage in patients affected by BED (51).

### Preconception Nutritional Profile in Adolescents and Women With EDs (See Tables 1, 2)

Healthy BMI prior to pregnancy is considered between 18.5 and 25 kg/m^2^ and current evidence suggests that poor or excessive gestational weight gain (GWG) are risk factors for perinatal complications. According to the Institute of Medicine(IOM) there are reference tables for correct GWG during pregnancy (see Table 1) (73–76).

**Table 1:** IOM weight gain recommendations during pregnancy (52).

**Weight gain recommendations during pregnancy**			
**Pre-pregnancy** **weight** **category**	**BMI** **g/m**^**2**^	**Recommended** **range of total weight (lb)**	**Recommended rates of GWG in the 2nd and 3rd trimester (lb)** **Mean range (lb/week)**
Underweight	<18.5	28–40	1 (1–1.3)
Normal weight	18.5–24.9	25–25	1 (0.8–1)
Overweight	25–29.9	15–20	0.6 (0.5–0.7)
Obese	≥30	11–20	0.5 (0.4–0.6)

**Table 2:** Studies about the effects of eating disorders on maternal nutritional status: pre-conception period and pregnancy.

**Author (year)**	**References**	**Aim of study**	**Type of study population**	**Maternal nutritional status**	**Key results**
Hadigan et al. (2000)	(53)	To evaluate the accuracy of diet history compared to observed food intake in women with AN, and healthy age-matched controls	Cross-sectional case-control AN = 30 Controls = 28 United States	Diet history is accurate to assess fat intake and macronutrient composition in patients with AN, and demonstrates significant micronutrient deficiencies in this population	- No differences were found between reported intake and observed intake for macronutrient composition, fat and energy in AN (1,602 ± 200 vs. 1,289 ± 150 Kcal, *p* < 0.05) - Micronutrient intake by diet history was highly correlated with observed intake in patients with AN (*p* < 0.05) - Based on reported intake, 50% of AN failed to meet the RDA for vitamin D, calcium, folate, vitamin B12, zinc, magnesium, and copper - 50% of controls failed to meet the RDA for vitamin D, folate, calcium, magnesium, iron, zinc, and copper - No correlation was found between reported intake of macro and micronutrients and energy in controls *p* < 0.05
Nova et al. (2004)	(54)	To assess the evolution of serum biochemical indicators of nutritional status in a 1-year follow-up of patients with restricting-type AN (rAN)	Longitudinal case-control AN = 14 Controls = 15 Spain	Ferritin and zinc levels were affected by nutrient requirements of anabolic processes during recovery of nutritional status	- Transferrin levels at admission were lower in rAN patients than in controls (250.9 ± 54.9 vs. 285.9 ± 21.0, *p* < 0.05) - Calcium and zinc showed significantly lower values in rAN when compared to the healthy subjects (at admission, Calcium 8.85 ± 0.37 vs. 9.56 ± 0.25, *p* < 0.01) - The mean serum level of phosphorus, calcium, zinc and iron in patients did not change during the follow-up (repeated measures ANOVA *p*-values, range 0.105 vs. 0.366) - Changes in ferritin and zinc showed significantly negative correlations with changes in anthropometrical parameters (Zinc correlation range −0.62 to −0.73, *p* < 0.05)
Misra et al. (2006)	(55)	To compare nutrient intakes of girls with AN with those of healthy adolescents	Cross-sectional case-control AN = 39 Controls = 39 United States	Girls with AN hadlower fat and higher fiber intakes than do healthy adolescents, which results in lower calorie intakes	- Dietary intakes of calcium, phosphorus, iron, zinc, copper, selenium, and sodium did not differ significantly between the girls with AN, and the control subjects (1,169 ± 88 vs. 981 ± 8; 1,333 ± 82 vs. 1,216 ± 77; 17.4 ± 1.4 vs. 15.0 ± 0.9; 10.9 ± 1.1 vs. 9.3 ± 0.5; 1.2 ± 0.1 vs. 1.1 ± 0.1; 92.7 ± 6.9 vs. 101.9 ± 6.0; 2,879 ± 220 vs. 2,997 ± 137, respectively) - Greater intakes of vitamins A, D, and K and of most of the B vitamins in the AN group, because of greater supplement use (13,823 ± 1,405 vs. 7,133 ± 917, *p* < 0.001; 11.3 ± 1.3 vs. 6.7 ± 0.9, *p* < 0.01; 167.5 ± 47.5 vs. 66.9 ± 7.1, *p* < 0.05, respectively)
Micali et al. (2007)	(23)	To determine the impact of pregnancy on ED symptoms	Longitudinal cohort study (ALSPAC) UK *N* = 12,254	Women with a recent ED continued to have some ED symptoms in pregnancy, but fewer compared to before pregnancy Although at a lower level, women with a past history of ED also had ED symptoms in pregnancy	- Women with a recent episode of ED dieted, used laxatives, reported self-induced vomiting, and exercised more than other groups during pregnancy, non-obese and obese controls (*p* < 0.05) with dieted OR = 5.1, 2.1–11.9, Used laxatives OR = 49.6, 16.1–152.5; self-induced vomiting OR = 51.9, 26.4–102.1, and high exercice OR = 1.8, 1.0–3.3. - Compared to non-obese controls, they were also more likely to report ED cognitions in pregnancy and their weight and shape concern scores remained high during pregnancy (report a strong desire to lose weight OR 6.1, 3.4–10.7, *p* < 0.001, they felt they had put on too much weight OR 2.5, 1.3–4.8, *p* < 0.01; they felt a loss of control over eating. OR 4.6, 2.5–8.6, *p* < 0.001 and felt a high concern about weight gain OR 2.4, 1.2–4.7, *p* < 0.01) - Compared to non-obese controls, Women with past ED were also more likely than controls to have some ED behaviors and/or concerns about weight gain (report a strong desire to lose weight OR 1.6, 1.3–2.0, *p* < 0.001, they felt they had put on too much weight OR 1.4, 1.1–1.7, *p* < 0.01; they felt a loss of control over eating. OR 1.3, 1.1–1.6, *p* < 0.01 and felt a high concern about weight gain OR 1.3, 1.0–1.6, *p* < 0.05)
Bulik et al. (2007)	(17)	To explore the course of broadly defined eating disorders during pregnancy	Longitudinal Norwegian Mother and Child Cohort Study (MoBa) *n* = 41,157	Pregnancy appears to be a catalyst for remission of some eating disorders, but also a vulnerability window for the new onset of broadly defined BED especially in economically disadvantaged individuals	-Proportions of individuals remitting during pregnancy were 78% (EDNOSP), 40% (BN purging), 39% (BED), 34% (BN any type), 29% (BN non-purging type). Additional individuals with BN achieved partial remission. Incident BN and EDNOS-P during pregnancy were rare
Crow et al. (2008)	(19)	To studies both eating behaviors and disordered eating cognitions in pregnant women with various ED diagnoses	Longitudinal cohort *n* = 42 United States	Eating disorder symptoms improved during pregnancy, but worsened postpartum	ED examination for restraint, shape concerns, weight concerns, binge eating, and purging diminished from prepartum to intrapartum, but returned to approximately baseline levels postpartum. Frequencies log-transformed mean(SD), Prepartum/intrapartum/postpartum Restraint 1.94 (0.26), 1.20 (0.24), 1.94 (0.26), *p* < 0.0001 Shape concern 2.68 (0.24), 2.21 (0.22), 2.76 (0.24), *p* < 0.0001 Weight Concerns 2.65 (0.24), 1.77 (0.22), 2.70 (0.24), *p* < 0.0001 Binge eating 0.03 (0.15), −0.36 (0.14), −0.15 (0.13), *p* = 0.0086 Purge frequency 0.04 (0.17), −0.03 (0.15), −0.10 (0.16), *p* = 0.0094
Siega-Riz et al. (2008)	(56)	To examine the nutrient and food group intake of women with EDs during pregnancy	Longitudinal cohort. Cross-sectional Norwegian Mother and Child Cohort Study (MoBa) Total *n* = 37,307	Women with BN or BED before and/or during pregnancy exhibit different dietary patterns	- Higher intakes of total energy, total mono-saturated and saturated fat, and lower intakes of folate, potassium, and vitamin C in women with BED before and during pregnancy compared to controls (*p* < 0.02). Mean ± SD Total energy 2.459 ± 30.2 vs. 2348 ± 4.6, Total MS 27.8 ± 0.4 vs. 25.6 ± 0.1, Total Saturated fat 34.2 ± 0.5 vs. 31.3 ± 0.1, respectively Folate 268.9 ± 3.8 vs. 273.4 ± 0.7, Potassium 4008.1 ± 49 vs. 4018.6 ± 7.6, Vitamin C 155.5 ± 3.7 vs. 167.5 ± 0.6, respectively - Higher total energy and saturated fat intake in women with incident BED compared to controls (*p* = 0.01) Total energy 2544.1 ± 41.2 vs. 2348.3 ± 4.6, Saturated fat intake 34.9 ± 0.6 vs. 31.3 ± 0.1, respectively
Swann et al. (2009)	(57)	To explore attitudes toward weight gain during pregnancy in women with and without EDs. To examine associations among weight-gain attitudes and actual GWG	Prospective population-based Norwegian mother and child cohort study (MoBa) *n* = 35,929	Women with EDs tend to experience weight-gain related worry during pregnancy. Early worry about gestational weight-gain may be aprecursor of high gestational weight gain	- The presence of an eating disorder was associated with greater worry over gestational weight gain AN Very Worried OR (95% CI) 49 (17–142), Somewhat worried OR 4 (95% CI) (1–15) BN Very Worried OR 62(95% CI) (41–92), Somewhat worried OR(95% CI) 7 (4–10) EDNOS-P Very Worried OR (95% CI) 80 (19–345), Somewhat worried OR (95% CI) 15 (3–65) BED Very Worried OR (95% CI) 7 (4–10), Somewhat worried OR (95% CI) 3 (3–4) - Women with BED who reported greater worry also experienced higher weight gains during pregnancy (17 kg vs. 16 kg in somewhat worried and 16 kg in not worried, *p* < 0.05)
Setnick (2010)	(58)	To assess the findings reported to date regarding micronutrient deficiencies and supplementation for patients with anorexia and bulimia	Narrative review	Patiens with ED showed micronutrients deficiencies due to low intake	Patients with EDs were found to have electrolyte disturbances, low levels of calcium, iron, zinc, vitamina B1, B6, B2, vitamin D, vitamin B12 and folate. Vitamina E and C deficiency was rare
Siega-Riz et al. (2011)	(59)	To examine the amount of weight of women with eating disorders AN, BN and BED gained during pregnancy and to evaluate the adequacy of total weight gain	Cross-sectional Norwegian Mother and Child Cohort Study *n* = 35,148	Women with AN had a lower risk of gaining weight inadequately while women with BN and BED were more likely to gain excessively weight during pregnancy	- Mean gestational weight gain for the entire sample was 2.5 (SD 0.02) kg at 17.0–20.1 weeks gestation, 9.3 (SD 0.03) kg at 27.4–29.7 weeks gestation and 15.0 (SD 0.03) kg at delivery. - Women with BN and BED gained significantly more weight on average than those with no EDs at each time point. BN 3.9 (0.3) vs. 2.5 (0.02), 10.9 (0.3) vs. 9.3 (0.03), and 16.6 (0.4) vs. 14.9 (0.03), at each respective time point, *p* < 0.001 BED 3.2 (0.1) vs. 2.5 (0.02), 10.2 (0.2) vs. 9.3 (0.03), and 16.6 (0.2) vs. 14.9 (0.03), at each respective time point, *p* < 0.001 - Women with AN had a lower risk AOR 5 0.65 (0.24, 1.72) of gaining inadequately while women with BN and BED were more likely to gain excessively, AOR 5 1.09 (1.01, 1.18) and 1.11 (1.08, 1.14), respectively
Micali et al. (2012)	(60)	To investigate the frequency of consumption of various food groups and quality of intake (macronutrient intakes) in a large general population cohort (ALSPAC)	Cross-sectional AN = 151 BN = 186 AN = BN = 77 Controls = 9,723	Despite some differences in food group consumption, women with lifetime ED had similar patterns of nutrient intake than healthy controls	- Women with lifetime ED scored higher on the “vegetarian” dietary pattern (OR 2.8, CI 95% 2.1–3.8) - No deficiencies in mineral and vitamin intake were evident in lifetime ED, although small differences were observed in macronutrient intakes across groups. All index groups had higher intakes of Mg and Se; women with AN and AN+BN of Fe and K and women with AN+BN of P, Ca, and Zn compared to unexposed women A lower sugar and non-milk extrinsic sugar and a higher polyinsaturated fat intake in women with lifetime AN and AN+BN. A higher fiber intake was also observed in women with lifetime AN, BN, and AN+BN. AN increased the risk for a high (>2,500 g/week) caffeine consumption in pregnancy (OR 2.6, 95% CI 1.4–4.8)
Micali et al. (2012)	(61)	To investigate adverse perinatal outcomes and GWG trajectories in women with lifetime (current/past) eating disorders: AN and BN	Longitudinal population-based birth cohort AN = 129, BN = 209, AN+BN = 100, Controls = 3,816 UK	Maternal lifetime ED is associated with few adverse perinatal outcomes. Differential GWG patterns in women with AN and BN are consistent with possible biological compensatory mechanisms aimed at protecting the fetus	- Maternal AN was positively associated with suspected fetal distress after adjusting for confounders OR 1.8; 95% CI 1.0–3.1, *p* < 0.05. No differences were found in mean birthweight, prevalence of a small-for-gestational-age, or premature birth. AN vs. BN vs. AN+BN vs. unexposed BW 3.481 g (480), 3,468 (575), 3,465 (586) vs. 3,439 (546) 0.8 vs. 4 vs. 2.1 vs. 3.5% 3.2 vs. 3.5 vs. 7.8 vs. 4.3% - Relative to unexposed women, women with AN had on average, a lower body weight regression coefficient −0.05 (95% CI −0.08 to −0.1) but a higher rate of weight gain, 0.07%, whereas women with BN had a higher body weight (regression coefficient 0.0007, 95% CI 0.00002–0.001) but a lower rate of weight gain (regression coefficient −0.0007, 95% CI −0.001 to −0.0001)
Velickovic et al. (2013)	(62)	To investigate association of vitamin D with BMD and BMI in EDs patients	Cross-sectional cohort *n* = 50 Australia	Low vitamin D and low BMI are associated with low BMD in EDs patients. Vitamin D measurement is appropriate in these patients	−18% prevalence of vitamin D deficiency below 50 nmol/L - Association between vitamin D and BMD T-score at the lumbar spine *p* = 0.029 (B = 0.206, 95% CI 0.120–0.293), femoral neck (*p* < 0.001) (B = 0.133, 95% CI 0.037–0.229) and total hip (*p* = 0.001) (B = 0.156, 95% CI 0.070–0.242) - No relation between vitamin D and BMI (B = −1.451, 95% CI −4.096–1.193)
Coker et al. (2013)	(63)	To examines changes in BMI and qualityof life related to eating disorders (QOLED) prior to, during and after pregnancyin both women with and without eating disorders	Longitudinal ED = 19 Controls = 159 Australia	Pregnancy is not associated with recovery from ED	The women with ED had significantly lower BMIs before, during and after pregnancy than non-ED. Mean difference (MD) and (SD) for all time points: pre-pregnancy 2.09 (0.86, *p* = 0.011), 1st trimester 2.12 (0.86, *p* = 0.023), 2nd trimester 2.37 (1.08, *p* = 0.043), third trimester 2.79 (0.94, *p* = 0.007), at 3 months 3.27 (0.71, *p* < 0.001), at 6 months 2.92 (0.74, *p* < 0.001), at 12 months after delivery 3.67 (0.67, *p* < 0.001). Both women with and without ED had significant weight gain in the second (ED *p* = 0.01 and non-ED *p* < 0.001) and third trimesters (ED *p* = 0.001 and non-ED *p* < 0.001) compared with pre-pregnancy. There were significant interactions between stage of pregnancy and ED status on global QOLED scores (Wald X^2^ = 25.40; d_f_ = 6, *p* < 0.001). These scores improved significantly during second (mean 8.19; SD 4.36, *p* = 0.017) and third trimesters (mean 8.80; SD 4.67, *p* = 0.033) compared with pre-pregnancy but varied after pregnancy, particularly among women with eatingdisorders. The QOLED scores for women with ED remained within the ED range throughout the study
Sabel et al. (2013)	(64)	Determine the prevalence of hematologic abnormalities in adults with severe AN	Retrospective *n* = 53 United States	Hematologic deficiencies are often present in patients with severe AN but resolves with nutritional rehabilitation	−83% of patients were anemic with only 3 (6%) having iron deficiency - 79% were leukopenic, 29% were neutropenic, 25% were thrombocytopenic, and 17% of patients developed thrombocytosis during their hospitalization
Zerwas et al. (2014)	(65)	To examine gestational and postpartum weight trajectories in mothers with and without EDs in the Norwegian Mother and Child Cohort Study (MoBa)	Longitudinal Cohort AN = 56 BN = 363 BED = 3,327 EDNOS = 69 Controls = 61,233 United States	Mothers with AN, BN, EDNOS and BED gained weight more quickly during pregnancy, and lost weight more quickly over the first 6 months postpartum than mothers without eating disorders	-Mothers with AN, BN, BED, and EDNOS had greater increases in BMI during pregnancy and greater decreases in BMI over the first 6 months postpartum - Women with AN shifted from the underweight BMI range before pregnancy to the normal weight range at 36 months postpartum: Mean BMI (SD) 18.2 kg/m^2^ (0.6) vs. 19.5 k/m^2^ (2.1)
Watson et al. (2014)	(8)	To validate previously published rates of remission, continuation, and incidence of broadly defined eating disorders during pregnancy	Summary of 19 studies based on the MoBa study Population-based pregnancy cohort	EDs during pregnancy were relatively common, occurring in nearly 1 in every 20 women, although almost all were cases of BED. Pregnancy was a window of remission from BN but a window of vulnerability for onset and continuation of BED	Pre-pregnancy prevalence estimates in the “validation sample” were 0–1% for AN, 1% for BN 3.3% for BED and 0.1% for purging disorder (EDNOS-P)
Gatti (2015)	(66)	To determine the prevalence of vitamin D deficiency in untreated patients with AN, and the role of vitamin D deficiency in bone metaboslism	Longitudinal cohort *n* = 89 Italy	There is an association between vitamin D and hip BMD values. Values of vitamin D above 20 ng/ml are recommended.	−16.9% had 25OH vitamin D levels <12 ng/ml, 36% <20 ng/ml and 58.4% <30 ng/ml - Patients with severe vitamin D deficiency (<12 ng/ml) presented BMD lower than that measured in groups with values over 20 ng/ml. BMD femur neck (732 ± 134 vs. 858 ± 106) BMD total hip (721 ± 116 vs. 871 ± 85) (*p* < 0.001 for trend) - PTH was higher and BMD of femoral neck and total hip were lower in patients with levels of vitamin D <20 ng/ml compared to patients with Vit D >20. PTH 39.1 pg/ml ± 21.1 vs. 27.5 pg/ml ± 12.9 8 *p* < 0.01) BMD femoral neck 755 mg/cm^3^ ± 111 vs. 879 mg/cm^3^ ± 104 (*p* < 0.001) BMD total hip 749 mg/cm^3^ ± 102 vs. 889 mg/cm^3^ ± 99 (*p* < 0.001)
Koubaa et al. (2015)	(67)	To investigate serum biomarkers of nutrition and stress in pregnant women with previous EDs	Longitudinal cohort ED = 37 Controls = 59 Sweden	Low maternal serum ferritin in women with previous AN may be of importance for impaired memory capacity in the offspring at 5 years of age	- Serum levels of ferritin in the women with previous AN, but not in those with a history of BN were significantly lower than in the controls (*p* < 0.01), and correlated strongly to impaired memory function in their children - Women with AN showed lower BMI and low GWG during pregnancy than BN or control group. Maternal BMI Mean (SD) 18.9 ± 2.9 vs. 21 ± 3.0 (*p* < 0.001) AN vs. BN and 18.9 ± 2.9 vs. 22.5 ± 2.8 (*p* < 0.01) AN vs. control group Maternal weight gain mean (SD) 10.0 ± 3.8 Kg vs. 12.9 ± 3.8 (*p* < 0.01) AN vs. BN and 10.0 ± 3.8 Kg vs. 12.2 ± 2.7 (*p* < 0.01) AN vs. control group
Jagielska (2016)	(68)	Review of the etiology, prevalence, course and treatment of bone mineralization disorders in AN	Narrative Review	AN has been associated with a high risk of osteoporosis and osteoporotic fractures at a young age. Further studies are needed to establish a standardized treatment for osteoporosis in AN women	- BMD values for osteopenia and osteoporosis were found, respectively, in 35–98% and 13–50% in women with AN - Densitometric assessment of BMD is recommended in all patients with AN and amenorrhea ≥12 months - Supplementation of vitamin D and adequate calcium intake are recommended - Improvement of BMD is observed after estrogen replacement and medroxyprogesterone in teenage girls and with bisphosphonates in adult women
Chiurazzi et al. (2017)	(69)	To compare the intake of energy and macro- and micronutrients in women with restrictive anorexia nervosa (rAN) and healthy controls	Longitudinal case-control AN = 13 Controls = 13 Italy	Intakes reported by rAN patients did not meet requirements for most micronutrients	- Mean intake of sodium, phosphorus and zinc was higher in controls compared to rAN (*p* < 0.01). Mean (SD): Na 1591 mg ± 306 vs. 729 ± 372 (controls vs. rAN) *p* < 0.001 Phosphorus 995 mg ± 189 vs. 736 ± 180 *p* = 0.02 Zinc 7.97 mg ± 1.48 vs. 4.82 ± 2.18 *p* < 0.001
Nguyen et al. (2017)	(70)	To investigate a diet quality score in pregnant women with EDs	Population-based cohort Netherlands *n* = 6,196	Higher quality score in EDs group after adjustment for socioeconomic and lifestyle factors	-Women with a history of EDs had a higher diet quality than women without a history of EDs (B 0.24 SD, 95% CI: 0.15; 0.33). Mothers with a history of EDs were less likely to breastfeed (unadjusted OR 0.68, 95% CI: 0.51; 0.93)
Achamrah et al. (2017)	(71)	To assess micronutrient status ina a large population of patients with AN	Retrospective Cross-sectional EDs: 153	Micronutrient status is often altered in AN patients, This mau contribute to neuropsychiatric dysfunction	- At least one trace element deficit was observed in almost half of patients; the most frequent was selenium deficit (40% of patients) - At least one vitamin deficit was observed in 45.7% of patients, mostly vitamin A and B9. Albumin, transthyretin and CRP were within normal range in most patients - No correlations were found between body composition and micronutrients status
Carlsson et al. (2018)	(72)	To investigate concentrations of total and free serum 25(OH)D in patients with AN and healthy controls	Longitudinal Case-control cases = 20 (27.6 ± 4.6 years) controls = 78 Sweden	Patients with AN did not showed vitamin D deficiency	- No differences in total or free S-25(OH)D levels: 80 ± 31 vs. 72 ± 18 nmol/L, and 6.5 ± 2.5 vs. vs. 5.6 ± 1.8 pg/ml, respectively, between cases and controls - In patients with AN bone mineral density (BMD) showed no correlation with total or free serum 25(OH)D

Few studies reported micronutrients status in patients with EDs with controversial results, because the majority evaluated dietary intake based on questionnaire reports and others assessed plasma levels. Cross-sectional and longitudinal case-control studies conducted on women of childbearing age with a history of AN determined a predominance of low intake of micronutrients like vitamin D, calcium, phosphorus, folate, sodium, magnesium, zinc, and copper. With regard to vitamin B12, results about the intake were contradictory: one study showed low intake in patients with AN characterized by high rate of vegetarians, but others did not find differences in intake when compared with the control group (53, 69). The limitations of these studies were the small sample sizes and the estimation of food portions, which were based on food questionnaire, since patients may underestimate or overestimate their nutrient intake. The high rate of vegetarianism among EDs women and consequently the lack of intake of meat and fish, is linked to a low source of iodine, iron, vitamins A, D, B12 and long chain omega-3 polyunsaturated fatty acids (77).

Another cross-sectional study did not find differences in dietary intake of minerals in adolescent girls with AN. In fact, the authors described a great intake of vitamins due to higher supplement consumption. However, these results were described in community-dwelling girls with AN, included in interventional and support strategies (55).

A review of literature conducted by Setnick (58), reported blood electrolyte disturbances in patients with EDs due to purging behaviors. Moreover, the author showed inadequate calcium intake, iron deficiency anemia, and zinc deficiency because patients restricted meat and practiced heavy exercise with consequent impaired absorption of elements. In contrast, vitamin A could be elevated in serum due to inadequate intake of other nutrients essential for vitamin A metabolism like dietary fats. Patients with EDs were found to have low ingestion of Vitamin B1, B2, and B6 because of restrictive eating patterns associated with alcohol consumption. Plasma deficiencies of Vitamin B12 and folic acid were also present in patients with EDs due to low intake, as judged by elevated plasma levels of homocysteine. Hematological deficiencies such as anemia, leucopenia, and thrombocytopenia are often present in patients with severe AN (54, 64).

Adolescence is a critical period for bone mass content, which is regulated by hormonal pathways. Estrogen and androgen release are decreased and cortisol increases in patients with EDs. Furthermore, conversion of free T3 and production of insulin-like growth factor-1 (IGF-1), are reduced. Such hormonal imbalances joined with nutritional deficiencies in vitamin D and calcium, decrease bone establishment, so 50% of adolescents with EDs displayed low mineral density (78). Young women affected by EDs were found to be vitamin D deficient (<50 nmol/L) and vitamin D levels predict low bone mineral density (BMD) in the lumbar spine, femoral neck and total hip. Weight gain was the most important factor in the improvement of BMD (62, 66). AN is associated with a high risk of osteoporosis and osteoporotic fractures at a young age (68). Conversely, Carlsson et al. (72) didn't find any differences in total and free 25 (OH) vitamin D in AN patients and healthy controls. A possible explanation could be that AN patients have a lower amount of adipose tissue, and vitamin D is a fat-soluble vitamin stored in fat tissue. If these patients present a deficit in fat mass, adipose tissue can't uptake vitamin D, resulting in increased levels. Nevertheless, this study was conducted in a specific population (Swedish), and vitamin D supplements were not registered, so it is difficult to generalize these results.

One of the most recent studies documenting nutritional status in 153 young women with AN found at least one trace element and one vitamin deficiencies in almost half of patients, the most frequent were selenium, vitamin A and B9, suggesting that micronutrient status is often disrupted in AN patients (71).

### The Impact of EDs During Pregnancy on Eating Patterns and Maternal Nutritional Profile (See Table 2)

Weight management during pregnancy is imperative for the health of offspring and it is linked to a balanced diet and to a regular physical activity (see Table S1). The majority of studies that report on eating behaviors in pregnant patients with EDs have found a high rate of remission of symptoms related to image concern, and decreased restrictive and purging attitudes (8, 19). These changes might be related to a sense of responsibility for fetal development, a different perception of their body during pregnancy, and greater support from family and clinicians (79). Other studies redact that pregnancy may trigger a relapse of EDs symptoms (23). Moreover, pregnancy may promote a remission of symptoms in patients with BN but it may generate the onset or continuation in BED patients (17). A recent systematic review showed that women with lifetime AN, BN, or AN+BN had similar nutritional patterns and similar intake of supplements as healthy women suggesting a remission of ED symptoms during gestation. This data only found greater energy and fat intakes in BED population (80).

A study conducted in a large UK general population cohort (ALSPAC) (60) included 414 patients and 9,723 controls and assessed eating patterns and nutrient intake in women with lifetime EDs during the third trimester of gestation using the Food Frequency Questionnaire (FFQ). The results showed that, in general, dietary patterns during pregnancy in women with EDs were similar to women from the general population. In relation to energy, carbohydrate, fat, and protein intake, no differences were found in the EDs group when compared to the control group. Nevertheless, they highlight that women with EDs consumed more soy products and plant-based food than controls and defined themselves as vegetarians. Moreover, women with AN and AN+BN showed high folate and potassium consumption; women with AN+BN had a higher intake of calcium, phosphorus, zinc, and vitamin C, and women with BN had higher vitamin E ingestion. They did not observe differences linked to macronutrient consumption. Interestingly, women with EDs consumed higher amounts of caffeine (>2,500 mg/week) than general population during pregnancy (81), probably due to its stimulating properties and to the desire to inhibit appetite.

Siega-Riz et al. (56) conducted a cross-sectional study in a large (*n* = 35,148) Norwegian Mother and Child Cohort (MoBa) and investigated food group intake by FFQ in pregnant women with BN and BED in the first half of pregnancy. Women with BED showed greater intake of total energy and total mono-saturated and saturated fat. As for micronutrient consumption, a small difference was found in women with EDs compared with controls. Furthermore, women with BED showed a lower intake of folate, potassium, and vitamin C related to lower intake of fruit and juices and a greater intake of fat (butter, margarines, and oils) and milk dessert. BN patients exhibited a lower intake of fatty meat but there was higher consumption of artificially sweetened drinks among women with BN and BED. Women with active BED also consumed more coffee than the control group in order to suppress appetite and to increase energy waste. The higher socio-economic status of such population may induce bias.

Nguyen et al. (70) investigated a diet quality score in pregnant women with EDs and found a higher quality score in EDs group after adjustment for socioeconomic and lifestyle factors. This result has been observed probably because women with EDs had a preference for food with health advantages (vegetables, skim milk) so they provide themselves a healthy diet out of concern about fetal development.

The strength of the studies described were the large population-based prospective design. The main limitations were related to self-reported ED history, because this may underestimate the ED, and to the assessment of portion size by FFQ, which is not precise.

In a recent longitudinal cohort study, Koubaa et al. (67) investigated pregnant nulliparous non-smoking women with a history of AN and/or BN vs. controls, analyzing biomarkers of nutrition during early pregnancy. Women with AN showed a higher prevalence of anemia, lower BMI, low GWG during pregnancy when compared to BN and the control group. Moreover, the authors found low plasma ferritin levels suggesting depleted iron stores. During pregnancy, mild anemia is a normal consequence of hemodilution and the requirement for iron rises because of increased fetal necessity and transfer across the placenta. Preconception iron stores determine the risk of iron deficiency anemia, so patients with EDs are at a greater risk for gestational anemia.

The main limitation of aforementioned studies is the lack of distinction between recent or ancient ED which may influence the nutritional profile of pregnant women with EDs.

Therefore, evidences support that pregnant women with EDs show sufficient quality in their diet demonstrating an improvement in ED symptoms during pregnancy. However, the ED subgroups exhibit differences related to the relapse of symptoms during pregnancy, micronutrient deficiencies and iron deficiency anemia, regardless to their intake and linked to malabsorption or impaired intestinal motility which should be close monitored. The increased demand for nutrient supply to maintain fetal growth might lead to the possible risk of unbalanced nutrient transfer, if required diet has not reached.

### The Impact of EDs During Pregnancy on GWG (See Table 2)

Studies carried out in longitudinal cohorts in Norway and the Netherlands exhibited higher and quicker GWG in women with EDs compared to controls. Micali et al. (60) described that women with AN had lower body weight but greater rates of GWG, whereas women with BN had higher body weight but lower rates of GWG.

Siega-Riz et al. (59) assessed GWG during pregnancy in the Norwegian Mother and Child Cohort Study in a cross-sectional approach. Authors demonstrated that women with AN had a lower risk of gaining inadequate weight during pregnancy, while women with BN and BED gained significantly more weight than those without an ED. Similar results were obtained by Zerwas et al. (65). These differences in GWG were linked to their eating pathologies, which persisted during pregnancy albeit more attenuate. Women with AN gained more weight than would be expected, probably because they exhibited the lowest pre-pregnancy weight, therefore evoking more concern for the unborn baby and undergoing partial remission of deregulated eating patterns. Gaining weight in women with ED might be protective for the increased demand of the fetus and it is the result of an improvement in ED behaviors and better control over purging patterns. The main limitations of these studies were the self-reported history of EDs and that GWG was based on maternal self-report which may underestimate it and induce bias. Moreover, large population samples included healthier patients with EDs than hospitalized women, and less severe symptoms were more likely to improve GWG.

Nonetheless, Koubaa et al. (82) found in a smaller sample, that anorexic pregnant women had considerably lower weight gain than controls (10.4 kg compared with 12.1 kg), demonstrating the persistence of restricted eating patterns and relapse of disordered eating during pregnancy. A possible explanation is that, in this study, patients with AN showed higher pre-pregnancy BMI if compared to the other studies, so the GWG was lower. On the other hand, women with BED who reported greater concern for GWG also experienced higher weight gain during pregnancy (57). Another study conducted in a small sample of ED patients found low GWG during every stage of pregnancy (63). This slow velocity of GWG may be explained by the most severe clinical manifestations in these samples of clinical patients with EDs, producing harmful effects on pregnancy and fetal outcomes.

### The Impact of EDs on Pregnancy Outcomes (See Tables 3, 4)

**Table 3:** Studies about the effects of eating disorders on pregnancy and fetal outcomes.

**Author (year)**	**References**	**Aim of study**	**Type of study. population**	**Maternal and fetal outcomes**	**Key results**
Blais et al. (2000)	(83)	To assess the impact of eating disorders (EDs: AN or BN) on pregnancy outcomes and the impact of pregnancy on cognitive and behavioral symptoms of EDs	Prospective study *N* = 54 United States	EDs women showed spontaneous abortion, therapeutic abortion and live birth	High rate of therapeutic abortion in EDs population
Franko et al. (2001)	(84)	To report obstetrical outcomes in a group of women with AN or BN	Prospective study Pregnant women with ED (AN and BN) *n* = 49 United States	Pregnant women with active EDs appear to be at greater risk for delivery by cesarean section and for postpartum depression	Pregnant women with EDs appear to be at greater risk for delivery by cesarean section (41 vs. 12%) and for postpartum depression (45 vs. 29%). Higher rate of birth defects (*n* = 3, 6.1%) No differences in rates of prematurity, Apgar score, or infant birth weight
Park et al. (2003)	(30)	To review evidence in genetic factors, pregnancy, the perinatal and postpartum period, infancy, and the early years, focusing on feeding and mealtimes, general parenting functions and growth	Review	ED pregnant women had higher rate of miscarriages, intra-uterine growth restriction, low infant birth weight, prematurity, perinatal mortality, lower Apgar score, and congenital abnormalities	- AN with low pre-pregnancy weight and low GWG have been associated with low infant birth weight, prematurity, perinatal mortality, lower Apgar score, congenital abnormalities - BN had a higher rate of miscarriages, intra-uterine growth restriction and congenital malformation - 5 possible mechanisms: genetic influences and gene-environment interactions, parental eating psychopathology may impinge directly on the child, disrupt general parenting functioning, learnt behavior, discordant marital, and family relationship
Sollid et al. (2004)	(85)	To determine the association of an ED diagnosed before pregnancy and a preterm delivery and/or the delivery of a low-birth-weight or small-for-gestational-age infant	Hospitalization records (more severe cases) Case-control prospective study (504 vs. 1,552) Danish population	ED pregnant women had a higher rate of low-birth-weight infant, preterm delivery, and SGA infants	- ED had Greater risk of Lower BW(OR, 2.2; 95% CI, 1.4–3.2), preterm delivery (OR, 1.7; 95% CI, 1.1–2.6), SGA (OR, 1.8; 95% CI, 1.3–2.4) - The risk of a low BW infant was twice as high in women with a previous ED compared with women with no such disorder - The risk of preterm delivery and a SGA infant was increased to 70% - Not mention to BMI, very severe cases
Kouba et al. (2005)	(82)	To examine pregnancy and neonatal outcomes in women with past or current eating disorders	Prospective case-control *N* = 97 Controls *n* = 68 Cases *n* = 49 (24 AN, 20 BN, 5 EDNOS) (Stockholm)	ED was associated to higher incidence of SGA, low birth weight, smaller head circumference and microcephaly	- SGA: 12 vs. 1%, *p* < 0.005 - Low birth weight (g): AN 3,210 ± 533 vs. controls 3,516 ± 515, *p* < 0.05 - Head circumference (cm): AN 33.7 ± 1.6, BN 33.7 ± 1.0, vs. controls 35.2 ± 1.6, both *p* < 0.001 - Microcephaly: ED 8% vs. controls 0%, *p* < 0.005 - IUGR: ED 8% vs. controls 0%, *p* = 0.07 - GA (weeks): ED 38.9 ± 1.8 vs. controls 39.2 ± 1.8, *p* = 0.043
Ekéus et al. (2006)	(86)	To examine birth outcomes and pregnancy complications in women with a history of AN	Prospective cohort study, Nationwide, Sweden Primiparous discharged from hospital with a diagnosisof AN vs. primiparous with no AN AN = 828,582 Controls = 827,582	Past history of AN was not associated with negative birth outcomes	- Mean birthweight (g): AN 3,387 vs. controls 3,431 g, *p* < 0.005. Non-adjusted by sex and GA - Small for gestational age (adjusted by sex and GA): no differences - Main birth outcome measures in women with a history of AN were very similar to the main population - Discrete lower BW
Newton and Chizawsky (2006)	(87)	To review the adverse fetal, birth, and maternal outcomes because of EDs. To enhance standard assessment practice and facilitate early intervention for the ED patient	Review	EDs had been associated to intrauterine growth retardation, premature birth, congenital anomalies, perinatal mortality, low birth weight, and microcephaly	ED had a higher risk of intrauterine growth retardation, premature birth, congenital anomalies (cleft lip and palate), perinatal mortality, low birth weight and microcephaly
Morgan et al. (2006)	(88)	To assess the impact of active BN in obstetric outcomes vs. quiescent BN	Retrospective Case-control study *n* = 122 Control = 89 UK	Active BN increase the risk of miscarriage and premature delivery	Active BN: - Higher % of unplanned pregnancies - Higher ORs for postnatal depression, miscarriage, and preterm delivery were 2.8 (95% [CI], 1.2–6.2), 2.6 (95% CI, 1.2–5.6) and 3.3 (95% CI, 1.3–8.8), respectively, GD (OR 5.7, 95% CI 1.2–26.6) and also hyperemesis
Micali et al. (2007)	(89)	To determine whether women with a history of eating disorders are at higher risk of major adverse perinatal outcome	Longitudinal cohort study (ALSPAC) UK AN = 171, BN = 199, AN+BN = 82 *n* = 10,636 unexposed	EDs had not an increased rate of preterm delivery. BN has been associated to miscarriage and AN to smaller birthweight babies	-BN was associated with miscarriage (relative risk ratio 2). Also, after adjusted for lifetime smoking and alcohol use, age and parity (OR 1,4 ((95% CI 1.1–2.0), *p* < 0.05) - AN had smaller babies (*p* < 0.05). It may be mediated by lower pre-pregnancy BMI and to a lesser extent by smoking in the second trimester of pregnancy. - Preterm delivery: AN 6.5%, BN 5.0%, AN+BN 4.9%, other psychiatric disorders 5.8%, general population 4.8%. After controlling for ethnicity, maternal age, and parity, Not differences between ED and general population. - Other psychiatric disorders group had higher rates compared with the general population (OR 1.3, 95% CI 1.0–1.8, *p* = 0.03). - No differences in preterm delivery
Bridget (2008)	(28)	To review the effect of ED in pregnancy and fetal outcomes	Review	ED had been associated to miscarriage, intrauterine growth retardation, premature birth, low birth weight, and microcephaly	BN: miscarriage AN: smaller babies, SGA, microcephaly, intrauterine growth restriction, and premature delivery (especially if the mother's body mass index was 20)
Bansil et al. (2008)	(90)	To describe trends in the prevalence of EDs among delivery hospitalizations in the United States from 1994 to 2004 and to compare hospital, demographic, and obstetrical outcomes among women with and without EDs	Retrospective *n* = 1,668 United States	Women with EDs are at increased risk of adverse pregnancy outcomes including fetal growth restriction, preterm labor, anemia, and genitourinary tract infections	Delivery hospitalizations with an ED were significantly more likely than those without an ED to have fetal growth restriction (odds ratio [OR] 9.08, 95% confidence interval [CI] 6.45–12.77), preterm labor (OR 2.78, 95% CI 2.10–3.69), anemia (OR 1.73, 95% CI 1.25–2.38), genitourinary tract infections (OR 1.66, 95% CI 1.03–2.68), and labor induction (OR 1.32, 95% CI 1.01–1.73)
Bulik et al. (2009)	(91)	To assess the association between EDs (AN, BN, BED, EDNOS-P) and pregnancy outcomes, controlled by confounding variables (GA, maternal age, income, education, parity, GWG) To assess the association with secondary outcomes (epidural, induction, placenta previa, non-vertex cephalic presentation)	MoBa study. Prospective cohort (*n* = 35,929), volunteered participation (42%). ED in the 6-months prior to or during pregnancy (AN *n* = 35, BN *n* = 304, BED *n* = 1,812, EDNOS-P *n* = 36) vs. referent group (*n* = 33,742)	ED was not associated to an increased rate of preterm delivery. BED is associated to a lower risk of small for gestational age babies and a higher risk of large for gestational age	- BED adjusted by gestational age, maternal age, income, education, and parity: Lower risk of SGA OR 0.65 (95% CI: 0.52, 0.8) *p* < 0.01, and after adjusting by smoking OR 0.63 (95% CI 0.51, 0.79) *p* < 0.01. Large for gestational age OR 1.2 (95% CI: 1.1, 1.4), *p* = 0.02, and after adjusting by smoking OR 1.2 (95% CI: 1.1, 1.4), *p* = 0.02 Preterm delivery OR 1.1 (95% IC: 0.92, 1.4) *p* = 0.65 - BED: higher% of LGA and c-section, lower% of SGA Not significant results on AN (low prevalence *n* = 35) - Preterm delivery adjusted by gestational age, maternal age, income, education, and parity: - AN: OR 0.63 (95% CI: 0.091, 4.3) *p* = 0.91 - BN: OR 0.78 (95% CI: 0.42, 1.4) *p* = 0.88
Bulik et al. (2010)	(42)	To assess unplanned pregnancy in AN patients	MoBA cohort AN = 62 vs. unexposed = 46,893	ED showed more unplanned pregnancies and induced abortion	RR 2.2 (95% CI, 1.64–2.72) of unintended pregnancies among AN Higher induced abortion (24.2 vs. 14.6%)
Pasternak et al. (2011)	(92)	To assess whether EDs (AN, BN or EDNOS) have an increased risk for adverse obstetric and perinatal outcomes	Retrospective Population-based study ED = 122 (AN = 41BN = 62 EDNOS = 19)vs. controls = 117,875 Israel	Eating disorders are associated with increased risk of adverse pregnancy outcomes	EDs patients were at risk of Low BW (OR 2.5, 95% CI 1.3–5.0), Preterm delivery (OR 2.2, 95% CI 1.4–3.6) and C-section (OR 1.9, 95% CI 1.3–2.9) Include severe cases
Eagles et al. (2012)	(93)	To compare pregnancy outcomes of women with and without a history of AN	Prospective case-control matched (1 case: 5 controls) by age, parity and year of birth (1965–2007) *n* = 804 Controls *n* = 670 women-−1,144 babies Cases (AN) *n* = 134 women-−230 babies Scotland	AN had a higher incidence of IUGR	AN were at risk of IUGR: RR 1.54, 95% CI 1.11–2.13
Linna et al. (2013)	(51)	To assess how eating disorders are related to reproductive health outcomes in a representative patient population	Retrospective EDs *n* = 2,257 Controls *n* = 9,028 Helsinki	EDs were associated with increased risk of induced abortion and miscarriage compared to controls	-Women with BED were more than 3 times as likely to have a miscarriage than controls [odds ratio (OR) 3.18; 95% confidence interval (CI) 1.52**–**6.66]. - Women with atypical BN, defined as those with one or more absent features of typical BN, were 44% more likely to have a miscarriage than controls (OR 1.44; 95% CI 1.02**–**2.04)
Solmi et al. (2014)	(94)	To quantify the effect of maternal AN (active or past) on birth weight	Systematic review (14 studies) Meta-analysis (9 studies) 1999–2012	AN was associated to smaller birthweight but with an important methodological heterogeneity between studies	Systematic Review: AN and other Eds were associated with smaller birthweight, differences found from −198 to −306 g. Nevertheless, the most of the studies don't adjust by sex and gestational age; after this adjustment, differences were smaller. - Meta-analysis (active or past AN): a standardized mean difference of weight: −0.19 kg (95% CI: −0.25, −0.15; *P* = 0.01) - Good evidence of heterogeneity in the studies (χ^2^ = 18.79, *P* = 0.016; *I*^2^ = 57.4%)
Linna et al. (2014)	(95)	To assess pregnancy, obstetric, and perinatal health outcomes and complications in women with lifetime ED	Prospective case-control population—based study *N* = 7,379 Controls *n* = 6,319 Cases *n* = 1,078 (AN = 302, BN = 724, BED = 52) Helsinki	AN and BN were associated with lower birthweight. AN was also associated with IUGR, preterm delivery and perinatal death. BN was also associated to lower Apgar score at 1 min and need of resuscitation. BED was associated to higher birthweight and to large for gestational age.	- AN and BN had significantly lower BW babies (mean 3,302 g [SD 562], adjusted *p* < 0.001 in AN, mean 3,464 g [563], adjusted *p* = 0.037 in BN, mean 3,520 g [539] in unexposed women) **-**BED had higher weight babies (mean 3,812 g [519], adjusted *p* < 0.001). - AN was significantly associated with anemia, slow fetal growth (OR 2.59, 95% CI 1.43–4.71), small for gestational age (OR 2.20, 95% CI 1.23–3.93) premature contractions, very premature birth (OR 4.59, 95% CI 1.25–16.87), and perinatal death (adjusted OR 4.06, 95% CI 1.15–14.35). **-**BN was associated with premature contractions (OR 2.2, 95% CI 1.17–4.14), resuscitations of the neonate (OR 2.12, 95% CI 1.18–3.79), and a lowAPGAR score (OR 2.31, 95% CI 1.34–3.98) - BED was associated with maternal hypertension (OR 4.32, 95% CI 1.64–11.36)
Koubaa et al. (2015)	(67)	To investigate serum biomarkers of nutrition and stress in pregnant women with a previous ED compared to controls and in relation to head circumference and early neurocognitive development of the offspring	Prospective case-control *N* = 96 Controls *n* = 59 Cases *n* = 37 (AN 20, BN 17) Sweden	EDs were associated to smaller head circumference even having the same serum levels of free thyroxine. AN group had a positive correlation between cortisol and head circumference	- Maternal serum levels of free thyroxine were similar between groups but correlated positively to reduced head circumference at birth of the children in the BN group (*r* = 0.48, *p* < 0.05), and with the same tendency in the AN group (*r* = 0.42, *p* = 0.07), but not in the controls (*r* = 0.006) - However, in the combined patient group, maternal free T4 correlated positively to head circumference at birth (rp = 0.36, *p* < 0.05), as in the BN group (rp = 0.48, *p* < 0.05), and with a similar tendency in the AN group (rp = 0.42, *p* = 0.07), - Cortisol levels were comparable between groups with the highest mean value in the control group. In the AN group only, there was a positive correlation between cortisol and head circumference of the offspring at birth (rs = 0.49, *p* < 0.05) - Head circumference (cm): AN 33.6 ± 1.6, BN 33.8 ± 0.88, control 35.2 ± 1.6, both *p* < 0.005 - Serum levels of IGF-I SD-score correlated positively with head circumference of the offspring at birth in the patient group (rs = 0.38, *p* < 0.05) but not in the separate groups
Triunfo and Lanzon (2015)	(96)	To review the associations between maternal undernutrition and obstetric risks	Literature review	Maternal undernutrition is associated to fetal growth restriction	Low intake of dietary nutrients determines a fetal growth restriction, may be due to an alteration in fetal hormones
Micali et al. (2016)	(97)	To investigate whether EDs are associated with lower size at birth, symmetric growth restriction, and preterm birth; and whether pregnancy smoking explains the association between AN and fetal growth	Prospective case-control *N* = 80,660 Controls *n* = 76,724 Cases *n* = 3,936 (AN 1,609, BN 1,693, AN+BN 634) UK	EDs, specially AN, had a negative impact in fetal growth and prematurity. Pregnancy smoking only partly explained the association between AN and adverse fetal outcomes	-IUGR: AN and AN+ BN: OR 1.6 [95% CI 1.3–1.8] - SGA: AN and AN+BN: OR 1.5 [95% CI 1.2–1.9). AN (15.5%) vs. controls (10.4%): OR 2.90 (95% CI 1.98–4.26). Lower birthweight was more associated with active AN than past AN, and it was lower than controls - Prematurity: Active AN + past AN: not differences on prematurity rates. Active AN: double rates of prematurity than past AN (7.51 vs. 4.11%). Active AN increased rates prematurity vs. controls (OR 1.77, 95% CI 1.00–3.12; *p* = 0.049). BN: not differences vs. controls
Kimmel et al. (2016)	(27)	To summarizes the literature on obstetric and gynecologic complications associated with EDs	Literature review	No consistent informations about fetal consequences of pregnancy women with eating disorders were found	Small samples studies report association between AN and increased risk of miscarriage, preterm birth, low birth weight infants, SGA infants, small head circumference, microcephaly, lower Apgar scores at 5 min, and greater risk of perinatal mortality. BN was associated with preterm birth and lower Apgar scores at 1-min. Bigger samples studies report contradictories results. Some studies affirm that women with ED (AN or BN) have increased rates of fetal growth restriction, preterm delivery, very premature birth, SGA, low birth weight, and perinatal death. While others cohort studies from different places of the world have consistently demonstrated no significant difference in adverse perinatal outcomes
Easter et al. (2017)	(98)	To investigate HPA axis regulation in women with Eds or their infants during the perinatal period	Prospective longitudinal study EDs = 47 Controls = 44 UK	During pregnancy women with ED had lower cortisol declines, suggestive of blunted diurnal cortisol rhythms. Postnatally, their infants also had a heightened response to stress	- Women with current ED (C-ED) had a significantly lower cortisol decline throughout the day compared to controls, in both adjusted and unadjusted analyses - Lower cortisol decline among women with a current ED was associated with higher levels of psychopathology during pregnancy - Women's cortisol awakening response, CRH and CRH-BP levels did not differ across the three groups - Infants' stress response was significantly higher among those in the C-ED group, although this effect was attenuated after controlling for confounders
O'Brien et al. (2017)	(37)	To study predictors of self-reported EDs and associations with later health events	Prospective case-control, cohort of sisters of women with breast cancer Controls *n* = 38,264 EDs *n* = 726 Puerto-Rican women	EDs were associated with higher rates of miscarriage and induced abortion	Miscarriage: OR 1.19 (95% CI 1.09–1.35) Induced abortion: OR 1.25 (95% CI 1.05–1.5)
Watson et al. (2017)	(99)	To determine if maternal ED increase risk of perinatal events	Retrospective Cohort *N* = 70,881 pregnancies in grandmother-mother-child triads. Controls (no ED) *n* = 65,586 Cases *n* = 5,295 (AN = 409, BN = 1,451, BED = 3,362, Purging disorder = 73). MoBa Cohort	EDs were associated with fetal effects, affecting specially the weight and the gestational age	-AN was associated with: low birth weight (*z*-score): RR 0.74 (95% CI 0.68, 0.82). SGA: RR 1.54 (95% CI 1.09, 2.17). Postmature: RR 0.47 (95% CI 0.33; 0.67). - BN: Induced labor: RR 1.21 (95% CI 1.07, 1.36). - BED: Birth weight (*z*-score): RR 1.07 (95% CI 1.05; 1.1). Length > p90: RR 1.2 (95% CI 1.13; 1.28). LGA: RR 1.19 (95% CI 1.14; 1.26)
Paslakis and de Zwaan (2018)	(29)	To review obstetrical and fetal consequences of ED and to present specific clinical recommendations	Review	EDs had negatives effects in obstetrical and fetal outcomes	Higher incidence of miscarriage and induced abortions, premature birth, low birth weight, low Apgar scores, and perinatal death
Eik-nes et al. (2018)	(100)	To identify associations between a lifetime ED (and obstetric outcomes. Adjusted for parity, maternal age, marital status, and year of delivery	Patient ED unit vs. Population-based study (The HUNT Study) from clinical patient register EDs *N* = 532 vs. unexposed *n* = 43,567	This study corroborates available evidence on the associations between maternal ED and adverse obstetric outcomes	AN had Higher rate of SGA (OR) 2.7, 95% CI 1.4–5.2. BN showed Higher rate of C- section OR 1.7 95% CI 1.1–2.5
Charbonneau and Seabrook (2019)	(101)	To investigate several adverse birth outcomes associated with Eds during pregnancy: miscarriage, preterm birth (PTB), low birth weight (LBW), SGA, and LGA To assess current ED and past ED	Narrative review (18 studies)	Of the 18 articles reviewed, EDs were associated with preterm birth and small-forgestational-age	- Eds were associated with preterm birth in 5/14 (36%) and SGA in 5/8 (63%) studies - AN increases the odds of a LBW baby, particularly when women enter pregnancy with a low BMI - BED is positively associated with having a LGA infant - BN was associated with miscarriage when symptomatic during pregnancy - Having a current ED increases the risk for adverse birth outcomes more than a past ED
Chan et al. (2019)	(102)	To assess adverse outcomes of EDs in pregnancy, from 1st trimester to 6 months postpartum. Assessment of covariates (BMI, psychological factors, anxiety and depressive symptoms) To assess the association between EDs and risk factors	Prospective longitudinal in a clinical population 1,470 Chinese pregnant women with EDs	Preterm delivery, Apgar scores and BW Disordered eating was assessed with the Eating Attitudes Test-26	- Only smoking before pregnancy was associated with disordered eating symptoms at the third trimester - LGA and low Apgar score were more likely to have mothers with higher levels of disordered eating at T2 and T3 (*p* < 0.01); - SGA was more likely to have mothers with higher levels of disordered eating at T2 (*p* < 0.005)
Sotodate et al. (2019)	(103)	To describe one possible fetal consequences of maternal vitamin k deficiency	2 cases reports	The maternal subclinical vitamin K deficiency due to an ED could induce a fetal intracranial hemorrhage	- EDs longer than 3 weeks could develop a maternal subclinical vitamin K deficiency and, consequently, a fetal intracranial hemorrhage - Pregnant women taking drugs inhibiting vitamin K metabolism or intestinal absorption. It must be recommended to take vitamin K from 36 gestational weeks until delivery onset

**Table 4:** Comparison among studies on pregnancy and fetal outcomes.

**Author. Ref**.	**Type of study/N**	**Pregnancy outcomes**	**Fetal outcomes**	**Strengths**	**Limitations**
Easter et al. (98)	Prospective longitudinal study Eds = 47 Controls = 44 UK	Significantly lower cortisol decline in women with current Eds (C-ED) throughout the day Lower cortisol decline among women with a C-ED was associated with higher levels of psychopathology	Infants' stress response was significantly higher among those in the C-ED group	Inclusion of past and current EDs	Small sample size AN and BN patients were grouped together Lack of pre-pregnancy cortisol assessment
Micali et al. (89)	Longitudinal cohort study (ALSPAC) UK AN = 171, BN = 199, AN+BN = 82, others ED = 1,166 *n* = 10,636 unexposed	Miscarriage in BN (OR 1,4 (95% CI 1.1–2.0), *p* < 0.05) No differences in preterm delivery	SGA in AN women explained by low maternal BMI (*p* < 0.05)	Large longitudinal sample size Adjusted for confounding factors (smoking, maternal age, parity) Similar maternal age Independent group with other psychiatric disorders	Self-reported EDs Self-reported GWG Higher smoking in AN Birth weight not adjusted for GA
Blais et al. (83)	Prospective study *N* = 54 United States	High rate of therapeutic abortion in EDs population		Classification for stage of severity	Small sample size Self-reported EDs No control group
Morgan et al. (88)	Retrospective Case-control study *n* = 122 Control = 89 UK	Active BN: - Higher % of unplanned pregnancies - Higher Ors for postnatal depression, miscarriage, and preterm delivery were 2.8 (95% CI, 1.2–6.2), 2.6 (95% CI, 1.2–5.6) and 3.3 (95% CI, 1.3–8.8), respectively, GD (OR 5.7, 95% CI 1.2–26.6) and also hyperemesis		Consideration of conflunding factors (alcohol, smoking, demographic differences) Primigravidae gestations	No control group Hospitalized population No differentiation between active and past BN
Sollid et al. (85)	Hospitalization records Case-control prospective study (302 women with 504 births vs. 900 women with 1,552 births) Danish population	Greater risk of preterm delivery (OR, 1.7; 95% CI, 1.1-2.6) in women with EDs	More incidence of Lower BW (OR, 2.2; 95% CI, 1.4–3.2), and SGA (OR, 1.8; 95% CI, 1.3–2.4) in ED population	Strict diagnostic criteria for EDs Large longitudinal sample size Consideration of conflunding factors (smoking, maternal age,) Primigravidae gestations	More severe cases No differentiation between AN and BN No information about GWG No information about obstetric complications No information about maternal nutrition
Bulik et al. (91)	MoBa study. Prospective cohort (*n* = 35,929), volunteered participation (42%). Cases *n* = 2,187 ED in the 6-months prior to or during pregnancy (AN *n* = 35, BN *n* = 304, BED *n* = 1,812, EDNOS-P *n* = 36) Referent group (*n* = 33,742)	-Preterm delivery adjusted by gestational age, maternal age, income, education, and parity: - AN: OR 0.63 (95% CI: 0.091, 4.3) *p* = 0.91 - BN: OR 0.78 (95% CI: 0.42, 1.4) *p* = 0.88 - BED: OR 1.1 (95% IC: 0.92, 1.4) *p* = 0.65 more C-section	BED had - lower risk of SGA babies OR 0.65 (95% CI: 0.52, 0.8) *p* < 0.01, and after adjusting by smoking OR 0.63 (95% CI 0.51, 0.79) *p* < 0.01 - More LGA OR 1.2 (95% CI: 1.1, 1.4), p = 0.02, and after adjusting by smoking OR 1.2 (95% CI: 1.1, 1.4), *p* = 0.02	Large longitudinal sample size Strict diagnostic criteria for EDs Strict definition of SGA and LGA Adjustment for GA, parity, obstetrical complications	Self-reported EDs Self-reported GWG Lack of pre-pregnancy BMI More healthier cases Specific population with high socioeconomic status
Pasternak et al. (92)	Retrospective Population-based study ED = 122 (AN = 41BN = 62 EDNOS = 19) vs. controls = 117,875 Israel	Eds were at risk of Preterm delivery (OR 2.2, 95% CI 1.4–3.6) and C-section (OR 1.9, 95% CI 1.3–2.9)	Eds were at risk of Low BW (OR 2.5, 95% CI 1.3–5.0),	Selected ED inclusion criteria Large sample size Exclusion of severe psychiatric disorders	More severe cases No differentiation between past and current EDs Not adjusted for confounding variables such as social class and smoking Inclusion of singleton and multiple pregnancies
Bansil et al. (90)	Retrospective *n* = 1,668 United States	Eds were more associated to preterm labor (OR 2.78, 95% CI 2.10–3.69), anemia (OR 1.73, 95% CI 1.25–2.38), genitourinary tract infections (OR 1.66, 95% CI 1.03–2.68), and labor induction (OR 1.32, 95% CI 1.01–1.73)	More fetal growth restriction (odds ratio [OR] 9.08, 95% confidence interval [CI] 6.45–12.77)	Large sample size Strict diagnostic criteria for EDs	No maternal BMI Not adjusted for confounding factors (maternal comorbidities, smoking, substance misuses, socioeconomic status)
Franko et al. (84)	Prospective study Pregnant women with ED (AN and BN) *n* = 49 United States	Pregnant women with EDs were at greater risk for delivery by cesarean section (41 vs. 12%) and for postpartum depression (45 vs. 29%). No differences in rates of prematurity	Higher rate of birth defects (*n* = 3, 6.1%) No differences in rates of prematurity	Strict inclusion criteria for EDs Division in symptomatic and non-syntomatic subgroups	Small sample size Absence of control group
Micali et al. (61)	Longitudinal population-based birth cohort AN = 129, BN = 209, AN+BN = 100, Controls = 3,816 Rotterdam	- Relative to unexposed women, women with AN had a lower body weight, regression coefficient −0.05 (95% CI −0.08 to −0.1) but a higher rate of weight gain, 0.07%, whereas women with BN had a higher body weight (regression coefficient 0.0007, 95% CI 0.00002–0.001) but a lower rate of weight gain (regression coefficient −0.0007, 95% CI −0.001 to −0.0001)	- Maternal AN was positively associated with fetal distress after adjusting for confounders OR 1.8; 95% CI 1.0–3.1, *p* < 0.05. No differences were found in mean birthweight, prevalence of SGA or premature birth. AN vs. BN vs. AN+BN vs. unexposed BW 3.481 g (480), 3,468 (575), 3,465 (586) vs. 3,439 (546) 0.8 vs. 4 vs. 2.1 vs. 3.5% 3.2 vs. 3.5 vs. 7.8 vs. 4.3%	Large longitudinal sample size Adjusted for confounding factors	Self-reported EDs Healthier population
Ekéus et al. (86)	Prospective cohort study, Nationwide, Sweden AN = 828,582 Controls = 827,582	- Main birth outcome measures in women with a history of AN were very similar to the main population	- Mean birthweight (g): AN 3,387 vs. controls 3,431 g, *p* < 0.005. Non-adjusted by sex and GA. - Small for gestational age (adjusted by sex and GA): no differences - Discrete lower BW	Strict inclusion criteria for EDs Large longitudinal sample size Adjusted for confounding factors Only primiparous	Exclusion of ED outpatients More severe EDs Strong clinical support may avoid perinatal complications
Koubaa et al. (82)	Prospective case-control Controls *n* = 68 Cases *n* = 49 (24 AN, 20 BN, 5 EDNOS) Stockholm		- SGA: 12 vs. 1%, *p* < 0.005 - Low BW (g): AN 3,210 ± 533 vs. controls 3,516 ± 515, *p* < 0.05. - Head circumference (cm): AN 33.7 ± 1.6, BN 33.7 ± 1.0, vs. controls 35.2 ± 1.6, both *p* < 0.001 - Microcephaly: ED 8% vs. controls 0%, *p* < 0.005 - IUGR: ED 8% vs. controls 0%, *p* = 0.07 - GA (weeks): ED 38.9 ± 1.8 vs. controls 39.2 ± 1.8, *p* = 0.043	Strict inclusion criteria for EDs No smoking, primiparous women High statistical power to detect microcefaly	Small sample size No differences beween past or recent EDs BW and head circumference not adjusted for GA or sex
Linna et al. (51)	Retrospective EDs *n* = 2,257 Controls *n* = 9,028 Helsinki	-Women with BED were more than 3 times as likely to have a miscarriage than controls (odds ratio (OR) 3.18; 95% confidence interval (CI) 1.52**–**6.66). - Women with atypical BN, were 44% more likely to have a miscarriage than controls (OR 1.44; 95% CI 1.02**–**2.04)		Strict inclusion criteria for EDs Large sample size	No information about remission or continuation of EDs
Linna et al. (95)	Prospective case-control population –based study *N* = 7,379 Controls *n* = 6,319 Cases *n* = 1,078 (AN = 302, BN = 724, BED = 52) Helsinki	- AN was significantly associated with anemia premature contractions, very premature birth (OR 4.59, 95% CI 1.25–16.87) - BN was associated with premature contractions (OR 2.2, 95% CI 1.17–4.14).-BED was associated with maternal hypertension (OR 4.32, 95% CI 1.64–11.36	AN and BN had significantly lower BW babies (mean 3,302 g [SD 562], adjusted *p* < 0.001 in AN, mean 3,464 g [563], adjusted *p* = 0.037 in BN, mean 3,520 g [539] in unexposed women) - BED had higher weight babies (mean 3812 g [519], adjusted *p* < 0.001). - AN was significantly associated with slow fetal growth (OR 2.59, 95% CI 1.43–4.71), small for gestational age (OR 2.20, 95% CI 1.23–3.93) and perinatal death (adjusted OR 4.06, 95% CI 1.15–14.35). - BN was associated with resuscitations of the neonate (OR 2.12, 95% CI 1.18–3.79), and a low APGAR score (OR 2.31, 95% CI 1.34–3.98)	Strict inclusion criteria for EDs Large sample size Singleton pregnancy Inclusion of obstetric complications	No differentiation among type of EDs More severe EDs No classification according to severity of ED No information about GWG
Watson et al. (99)	Retrospective Cohort *N* = 70,881 pregnancies in grandmother-mother-child triads. MoBa cohort Controls (no ED) *n* = 65,586 Cases *n* = 5,295 (AN = 409, BN = 1,451, BED = 3,362, Purging disorder = 73).	- BN: Induced labor: RR 1.21 (95% CI 1.07, 1.36)	-AN was associated with: low birth weight (*z*-score): RR 0.74 (95% CI 0.68, 0.82). SGA: RR 1.54 (95% CI 1.09, 2.17). Postmature: RR 0.47 (95% CI 0.33; 0.67). - BED: Birth weight (*z*-score): RR 1.07 (95% CI 1.05; 1.1). Length > p90: RR 1.2 (95% CI 1.13; 1.28). LGA: RR 1.19 (95% CI 1.14; 1.26)	Large sample size Inclusion of grandmother-mother-child triads Inclusion of GWG assessment	Self-reported EDs No adjustment for confounding factors
Micali et al. (97)	Prospective case-control *N* = 80,660 Controls *n* = 76,724 Cases *n* = 3,936 (AN 1,609, BN 1,693, AN+BN 634) UK	-Prematurity:Active AN + past AN: not differences on prematurity rates. Active AN: double rates of prematurity than past AN (7.51 vs. 4.11%). Active AN increased rates prematurity vs. controls (OR 1.77, 95% CI 1.00–3.12; *p* 0.049). BN: not differences vs. controls	- IUGR: AN and AN+ BN: OR 1.6 [95% CI 1.3–1.8]. - SGA: AN and AN+BN: OR 1.5 [95% CI 1.2–1.9). AN (15.5%) vs. controls (10.4%): OR 2.90 (95% CI 1.98–4.26). Lower birthweight was more associated with active AN than past AN, and it was lower than controls	Large longitudinal sample size Adjusted for confounding factors (smoking, maternal age, alcohol misuse) Analysis of past and current EDs	Self-reported EDs Self-reported GWG
Eik-nes et al. (100)	Patient ED unit vs. Population-based study (The HUNT Study- Norway) from clinical patient register Cases = 532 Unexposed *n* = 43,567	BN showed Higher rate of C- section OR 1.7 95% CI 1.1–2.5	AN had Higher rate of SGA (OR) 2.7, 95% CI 1.4–5.2	Large sample size Strict inclusion criteria for EDs	No adjustment for maternal socioeconomic status, BMI or smoking
Eagles et al. (93)	Prospective case-control matched (1 case: 5 controls) by age, parity and year of birth (1965–2007) *n* = 804 Controls *n* = 670 women- 1,144 babies Cases (AN) *n* = 134 women-−230 babies Scotland		AN were at risk of IUGR: RR 1.54, 95% CI 1.11–2.13	Large longitudinal sample size Strict inclusion criteria for EDs Adjusted for confounding factors (maternal smoking, socioeconomic status, BMI)	Only AN patients
Koubaa et al. (67)	Prospective case-control *N* = 96 Controls *n* = 59 Cases *n* = 37 (AN 20, BN 17) Stockholm	- Cortisol levels were comparable between groups with the highest mean value in the control group. In the AN group only, there was a positive correlation between cortisol and head circumference of the offspring at birth (rs = 0.49, *p* < 0.05)	- Maternal serum levels of free thyroxine were similar between groups but correlated positively to reduced head circumference at birth of the children in the BN group (*r* = 0.48, *p* < 0.05), and with the same tendency in the AN group (r = 0.42, *p* = 0.07), but not in the controls (r = 0.006)	Strict inclusion criteria for EDs Singleton, spontaneous pregnancies No smoking	Small sample size AN had minor BMI
		- Serum levels of IGF-I SD-score correlated positively with head circumference of the offspring at birth in the patient group (rs = 0.38, *p* < 0.05) but not in the separate groups	- However, in the combined patient group, maternal free T4 correlated positively to head circumference at birth (rp = 0.36, *p* < 0.05), as in the BN group (rp = 0.48, *p* < 0.05), and with a similar tendency in the AN group (rp = 0.42, *p* = 0.07) - Head circumference (cm): AN 33.6 ± 1.6, BN 33.8 ± 0.88, control 35.2 ± 1.6, both *p* < 0.005		
O'Brien et al. (37)	Prospective case-control, cohort of sisters of women with breast cancer Controls *n* = 38,264 Eds *n* = 726 Puerto-Rican women	Miscarriage: OR 1.19 (95% CI 1.09–1.35) Induced abortion: OR 1.25 (95% CI 1.05–1.5)		Large longitudinal sample size	Self-reported EDs High proportion of well-educated and non-hispanic women
Chan et al. (102)	Prospective longitudinal in a clinical population 1,470 Chinese pregnant women with EDs	Only smoking before pregnancy was associated with disordered eating symptoms at the third trimester (T3)	LGA and low Apgar score were more likely to have mothers with higher levels of disordered eating at the second trimester (T2) and T3 (*p* < 0.01); - SGA was more likely to have mothers with higher levels of disordered eating at T2 (*p* < 0.005)	Large longitudinal sample size	Self-reported EDs No diagnosis in the same stage of pregnancy Some questionnary missing

#### Endocrine Manifestations

EDs women display low levels of GnRH and consequently decreased levels of LH and FSH due to hypothalamic dysfunction. Moreover, even if in normal pregnancy leptin levels increase (104), low levels of leptin and peptide YY, both anorexic hormones, and high levels of ghrelin, an orexigenic peptide, have been described in women with EDs. The suppressed gonadotropin secretion in AN is associated with low T3 levels and low TSH, suggestive of a hypothalamic origin of suppressed thyroid function. Cortisol and CRH levels have been found to be elevated in women with AN and BN (105). GH levels are increased while IGF-1 levels decreased, demonstrating a GH resistant status as an adaptive mechanism to starvation. Most studies have also found increased levels of adiponectin in AN, which is inversely related to BMI and is associated with increased insulin sensitivity seen in AN women (106). Easter et al. (98) assessed HPA axis regulation in women with EDs. They observed different patterns of circadian salivary cortisol in pregnant patients, such as low morning cortisol levels, suggesting a blunted response of cortisol during the day. A possible explanation may be a decrease in HPA axis response due to a pre-gestational prolonged period of hypercortisol exposure in women with EDs. Moreover, this may be triggered by higher symptoms of anxiety and stress during pregnancy. The strength of this data was the longitudinal design and the inclusion of past and recent ED women. The limitations included that AN and BN patients were grouped together, the small sample size, and the lack of pre-pregnancy cortisol assessment. Further less, as depression or anxiety are multifactorial pathologies, it is difficult to establish a correlation with cortisol levels.

Hormonal changes might play a role in the remission patterns of EDs during gestation. It has been hypothesized that increased production of dihydroepiandrosterone (DHEA) by the placenta in patients with AN may counteract the adverse effects of cortisol, increasing the rate of remission during pregnancy (79).

#### Pregnancy Outcomes

Recent studies focused on pregnancy outcomes in women with EDs showed controversial findings. The main studies on ED population-based sample and clinical ED patients highlighted an increase in spontaneous abortion, particularly with BN (88), and also a high incidence of preterm delivery (42, 51, 83, 88, 89). These adverse outcomes may be promoted by multiple variables such as stress, hormonal imbalance, low leptin levels and conditions like PCOS. For example, low leptin levels have been associated with the risk of miscarriage, preterm delivery, and poor fetal growth (104).

The association between EDs and preterm delivery is controversial, and it was found more strongly related to hospitalized patients. Sollid et al. (85) designed a longitudinal case-control study, comparing pregnant women with EDs with healthy controls. They observed a higher risk of preterm delivery and small for gestational age (SGA) babies in the women with EDs but they analyzed more severe cases and they did not differentiate between AN and BN. Bulik et al. (91) found a higher rate of smoking, preterm delivery, and greater risk of delivery induction only in patients with BED. Additionally, in the BED population there was a higher risk to deliver a large for gestational age (LGA) newborn and a higher rate of cesarean section. Conversely BED women had lower rate of SGA probably because they gained more GWG. It is important to mention that the data was based on maternal self-report and this population had good socioeconomic status. Pasternak et al. (92) performed a retrospective study obtaining a higher rate of preterm delivery in more severe diagnosed ED women. This finding was associated with low pre-pregnancy BMI, low GWG, and higher rate of fertility treatment in the ED group. They also found a greater rate of cesarean section in BN and BED groups. Other retrospective study found similar results about the association between EDs and obstetric complications with no links to cesarean section (90). However, this study did not take into account confounding factors such as smoking and socioeconomic status. Conversely, Micali et al. (89) didn't find any association between EDs and preterm delivery although this study included a large longitudinal community cohort with less severe disease.

Franko et al. (84) reported greater risk for cesarean birth and postpartum depression in a group of women with AN and BN but no differences in the rate of prematurity. The limitations of these data were the absence of a control group, and the small group size. Neither significant differences in preterm delivery (61, 86), nor an increased risk of gestational diabetes (GD), hypertension, pre-eclampsia, induction of labor, instrumental delivery or postpartum bleeding were found in other prospective population-based studies in EDs women.

One potential explanation for these inconsistent results may be due to the inclusion criteria: in the population-based study, participants required only self-reported ED, whereas many clinical studies selected patients with EDs on the basis of hospitalization and with a more actively ED. Moreover, GWG improved faster in Norwegian and Dutch cohorts demonstrating the concern and support by caregivers to ED women and this may explain the lack of adverse outcomes.

Recent studies found a strong causal association between preterm delivery, fetal growth restriction, and low pre-pregnancy BMI, malnutrition and a low proportion of weight gain during pregnancy. This association remains after excluding pathologies such as diabetes and hypertension (107). A recent study conducted in a Norwegian population corroborated that higher diet scores, reflecting healthy dietary behaviors in the pre-pregnancy period and during pregnancy, prevent preterm delivery (108). Maternal stress in pregnancy and consequential high levels of CRH may also influence the timing of delivery and this could elucidate the risk of preterm labor in women with lifetime EDs (109). Furthermore, altered maternal mood and anxiety may damage the intrauterine environment, enhancing the risk of obstetric adverse outcomes (110, 111).

Although the association between EDs and poor obstetric outcomes is multifactorial, the severe of disease, the persistence of symptoms, low pre-pregnancy BMI and poor GWG, joined with environmental factors such as social dysfunction, potential stressors, smoking, drugs consumption and concomitant psychiatric disorders may worsen pregnancy evolution. However, a strong caregivers' and psychological support may avoid obstetric complication displaying normal pregnancy evolution.

Of note, a current ED increases the risk of adverse outcomes during pregnancy more than a past ED (101).

### The Impact of EDs on Fetal Outcomes (See Tables 3, 4)

Available studies postulate the potential association between maternal EDs symptoms during pregnancy and adverse fetal outcomes. Fetal development is intrinsically linked to balanced maternal nutrition, correct placental supply, and fetal growth factors. Maternal malnutrition may affect fetal development through the absence of nutrients, which are essential for fetal growth, modifications of placental function, and epigenetic changes in the fetal genome. Furthermore, maternal stressors may alter the imprinting of fetal regulatory elements (112). According to the theory of “early life programming,” an altered intrauterine environment may predispose the offspring to the risks of developing chronic diseases later in life (113).

Not all EDs types (AN, BN, BED, and ENDOS) have been associated with the same fetal risks and not all studies evaluated independently each type of ED. Therefore, the conclusions obtained from the different studies and reviews are not consistent.

#### Small for Gestational Age (SGA) and Intrauterine Growth Restriction (IUGR)

Population-based studies have shown that neonates of pregnant women with EDs are at higher risk of lower birth weight (BW) than those of healthy pregnant women. Although they included large sample size, one important limitation of these types of studies was the classification of EDs based on self-reported questionnaires. Furthermore, it is important to highlight that a large number of studies did not use the proper definition of SGA (weight percentile <10 adjusted for gender and gestational age) to evaluate neonates. For example, the study of Micali et al. (89) showed an association between AN and lower BW. This large sample size included more than 1,600 pregnant women who were classified as EDs with psychiatric disorders (*n* = 1,166), BN (*n* = 199) AN (*n* = 171), and AN+BN (*n* = 82). The data was adjusted by parity, maternal age, and smoker habits but the proper definition of SGA was not applied. The study of Watson et al. (99) also found a strict association between EDs and low BW and concluded that maternal EDs increase the risks of pregnant and neonatal complications, even when there is a correction for perinatal outcomes in the previous generation. In a similar line, Micali et al. (97) observed a higher odds of SGA (defined as birthweight <10th percentile) in both AN and BN (OR 1.6, 95% CI 1.3–1.8; OR 1.5, 95% CI 1.2–1.9, respectively) being more associated with active AN than with past AN.

Furthermore, longitudinal prospective studies conducted in Danish, Swedish, and Norwegian populations among hospitalized pregnant women diagnosed with ED according to strict criteria reported a higher incidence of low BW and SGA (82, 85, 95, 100). However, the women included in these studies were hospitalized, so they represented the most severe cases. The main limitation of Kouba's study (82) was the reduced sample size and the lack of differentiation between past or current ED. The main limitation of the study of Eik-nes et al. (100) was that, although they registered smoking and socioeconomic status, they did not consider these data to adjust the results. Recent systematic reviews (27, 28, 87, 94) supported these findings indicating that EDs are associated with low BW.

Conversely, other studies conducted in pregnant women with a clinical diagnosis of EDs, did not find differences in the association among EDs and low birth weight or the incidence of SGA (84, 86). Nevertheless, these studies included women who required hospitalization, and pregnant patients were strictly monitored and consequently this may reduce the number and severity of perinatal complications. Moreover, the study of Franko et al. (84) did not include a control group and Ekeus et al. did not report GWG, which could be a protective factor for impaired fetal growth.

With regard to IUGR, several studies corroborated the higher incidence in pregnant women with EDs, if compared to general population. Bansil et al. (90) analyzed a retrospective cohort of pregnant women with 1,668 cases of strictly classified EDs, observing an increased risk of IUGR (OR 9.08 95% CI 6.45–12.77). However, in this study the outcomes were not adjusted for maternal BMI, neither for confounding factors such as maternal comorbidities, tobacco habit, substance misuses and socioeconomic status. Eagles et al. (93) found similar conclusions in a case-control study with a larger sample size evaluating pregnant women with a clinical diagnosis of AN (134 cases), adjusting the results for confounding factors such as maternal BMI, smoking, and social class. Indeed, Kouba et al. (82) did not find those differences, but the study was conducted in a small sample size.

Therefore, we can state that generalization of these results is limited because of the diversity of study designs, the inclusion of different populations with wider range of severity, or small sample sizes. Nonetheless, updated literature endorses the notion that EDs during pregnancy may increase the risk of having a baby with low BW or SGA. The severity of EDs and an active ED have been associated with more risk of IUGR. Moreover, the strongest relationship between EDs and low BW was found in patients with low pre-pregnancy BMI and/or low GWG. In the aforementioned studies, the mean maternal body mass index was lower in the exposed than in the unexposed group. Smoking, also may interact in the delivery of low BW babies. Nevertheless, the majority of such studies didn't stratified their results for BMI categories and few studies reported GWG or nutritional status of the mother (89, 95, 99).

#### Large for Gestational Age (LGA)

Two case-control studies, both with more than 1,500 cases of EDs, concluded that pregnant women with an ED (BED subtype) had a higher risk of having LGA neonates (91, 99). Even more, they showed a strong association between BN or BED and gestational diabetes, which may clarify the greater incidence of LGA. Watson et al. (99) also found in BED patients a high incidence of neonates with length over the 90th percentile. The main weakness of these studies were that ED diagnosis was self-reported and in the study of Watson et al. statistical analysis was not adjusted for possible confounder factors (99).

The studies by Linna et al. (95) and Watson et al. (99) also showed that pregnancies in women with BED were more likely to be prolonged. However, they did not obtain the same magnitude of estimated risk (95, 99).

Additionally, a recent research found that women with BED had higher intake of saturated fat during pregnancy, which may explain the relationship between BED and larger size of newborns (56). Women with BED were also found to gain more gestational weight during pregnancy, affecting the fetal size (61).

#### Perinatal Mortality and Lower Apgar Score

Few data evaluated Apgar score and perinatal mortality in women with EDs reporting contrasting results so there is a lack of evidence to state that EDs is a risk factor for perinatal morbidity and mortality. In the case-control study of Linna et al. (95) neonates of pregnant women with BN exhibited lower Apgar at minute one and they needed more resuscitation in delivery room, but the most severe cases with binge and purge episodes were included in this study, increasing the risk of bias. Conversely, in the cohort of patients with EDs studied by Franko et al. (84), no differences were found in the Apgar score according to the diagnosis of AN or BN in pregnant women. However, this study was performed in a small sample size (only 49 EDs patients) and the results must be interpreted carefully because ED groups were not compared to a control group.

Regarding perinatal mortality, the case-control study of Eagles et al. (93) did not report increased risk in neonates of pregnant women with AN, but the study was conducted in a specific population with higher GWG which may mitigate the poor outcomes associated to EDs. Nevertheless, in the literature, the relationship between perinatal mortality and EDs has been widely reviewed (30, 87). Therefore, newborns of pregnant women with EDs are at risk of perinatal mortality if compared to general population. The relapse and exacerbation of ED symptoms during pregnancy is the most evident mechanism linked to perinatal mortality.

#### Lower Head Circumference(Microcephaly), Malformations, Intraventricular Hemorrhage

Two case-control studies showed lower head circumference and a higher incidence of microcephaly in neonates of pregnant women with EDs. Kouba et al. (82) were the first to describe this notion, considering that it could be due to high levels of maternal cortisol produced by stress, especially in pregnant women with AN who had a BMI <20. Another subsequent study by Koubaa et al. (67) concluded that neonates of primiparous and no smoking pregnant women with EDs, showed better head circumferences, even with the same serum levels of thyroxin, a hormone which correlated positively to head circumference. The authors concluded that, in pregnant women with AN, there was a correlation between maternal cortisol levels and neonatal cephalic perimeter. In three previous reviews on the fetal repercussions of maternal EDs, a correlation with lower head circumference was found; however, these results were observed in small sample sizes, and in the most severe ED patients but not in larger studies (27, 28, 87).

With regard to congenital malformations the reviews of Park et al. (30) and Newton et al. (87) stated that children of pregnant women with EDs showed increased incidence, but these reviews included previous studies dating back in 80's and 90's. Only Newton et al. detailed that these malformations were cleft lip and cleft palate (87).

Eight cases of prenatal intraventricular hemorrhage have been described, attributable to maternal vitamin K deficiency secondary to poor intake, or intestinal malabsorption. Placental transfer of vitamin K is scarce, and it is estimated that its concentration in cord blood is 1/30 with respect to maternal plasma levels, so a severe deficiency has been found in malnourished mothers (103).

Therefore, actually the link among EDs during pregnancy and microcephaly or malformations need more controlled studies to corroborate it, including the correction for several confounding factors such as maternal drugs, alcohol misuse, smoking, or severe maternal malnutrition.

#### Possible Causes for Poor Fetal Outcomes

Some studies tried to find a positive association between mothers with EDs and adverse pregnancy outcomes, impaired fetal growth, malformations, and poor prognosis in their offspring. In accordance with the theory of “fetal programming,” the nexus between nutrition and stress in pregnant women with EDs triggers the activation of maternal and fetal HPA axis, increasing levels of maternal CRH (96). As consequence, higher levels of glucocorticoids in fetal circulation have an impact on fetal programming and neural development, and are associated with low birth weight (114, 115). Smoking is frequent in AN patients, linked to the desire to control weight and appetite. Nicotine release appetite suppressors in the brain (serotonin, dopamine, and norepinephrine), which are also associated with impaired fetal growth (102, 115). In addition, the use of laxatives, diuretics, and appetite suppressants have been described to cause possible teratogenic complications (85). If nutritional deficiencies such as low iron or folate levels, low fish intake which is a source of iodine, L-PUFA and vitamins are mixed with maternal stressors, the risk of neural tube defects (67, 115), small head circumference and impaired cognitive development increases (116, 117). Furthermore, thyroid function and IGF-1 axis, essential for fetal growth, may be compromised in pregnant women with EDs (67).

The inconsistent of several studies is probably due to the difference in the severity of illness between the clinical vs. the population-based sample, finding a stronger association of poor perinatal outcomes among the most severe cases of EDs. Moreover, the presence of confounding factors were not taken into account in some studies, such as maternal age, severe psychiatric pathology, maternal BMI, or toxic habits like tobacco, drugs, or alcohol. Furthermore, the majority of the studies didn't differentiate between past or active EDs.

### Epigenetic Modifications: Association With EDs and Nutritional Deficits During Pregnancy (See Table 5)

Barker's hypothesis defines fetal programming as the process of fetal adaptation to the environmental conditions during development (113). Therefore, EDs or a specific nutritional deficiency during pregnancy produce severe alterations in maternal and fetal epigenetic profiles (124). Epigenetic mechanisms as the DNA methylation of CpG islands, the Histone tail modifications by enzymes as well as the expression control by microRNAs regulate the genomic imprinting, modulating cellular differentiation and organogenesis during fetal development (125).

**Table 5:** Studies about the effects of eating disorders on epigenome.

**Author (year)**	**References**	**Aim of study**	**Type of study. Population**	**Epigenetic changes**	**Key results**
Frieling et al. (2007)	(118)	To analize the global methylation pattern in women diagnosed with anorexia nervosa and bulimia nervosa To study candidate genes which expression is altered by nutritional deficits associated to anorexia or bulimia	Cross-sectional case-control in women Controls: 30 AN: 22 BN: 24	There are differences in the methylation pattern between patients with anorexia and controls in whole blood samples *SNCA* gene promoter is hypermethylated in anorexia patiens	Global DNA hypomethylation in women with anorexia nervosa DNA hypermethylation of the alpha synuclein promoter No differences in the global methylation status of women with bulimia
Frieling et al. (2010)	(119)	To study candidate genes which expression or methylation pattern are altered in women with anorexia or bulimia compared to control population	Cross-sectional case-control in women Controls: 30 AN: 22 BN: 24	*SLC6A3/DAT, DRD2*, and *DRD4* genes were analized in whole blood samples of anorexia and bulimia patients No methylation differences were observed for DRD4	*SLC6A3/DAT* and *DRD2* are hypermethylated in patients with AN In women with BN *SLC6A3/DAT* is also hypermethylated
Ehrlich et al. (2012)	(120)	To study candidate genes which expression or methylation pattern are altered in women with anorexia compared to control population	Cross-sectional case-control in women Controls: 54 AN: 40	*POMC* gene was analized in whole blood samples of anorexia patients	No methylation differences were observed
Steiger et al. (2013)	(121)	To study candidate genes which expression or methylation pattern are altered in women with bulimia compared to control population	Cross-sectional case-control in women Controls: 32 BN: 64	*GR* gen was analized in whole blood samples of bulimia patients	No methylation differences were observed
Tremolizzo et al. (2014)	(122)	To analize the global methylation pattern in women diagnosed with anorexia nervosa	cross-sectional case-control in women Controls: 13 AN: 32	There are differences in the methylation pattern between patients with anorexia and controls in whole blood samples	Global DNA hypomethylation in women with anorexia nervosa
Hoyo et al. (2011)	(123)	Effects of folic acid supplementation using Pyrosequencing to measure methylation at two *IGF2* DMRs	Prospective study in umbilical cord blood leukocytes, measuring folic acid intake before and during pregnancy in 438 pregnant women	Methylation at the *H19* DMR decreased in infants born to women reporting folic acid intake compared to infants born to women with no folid acid intake during pregnancy	The use of folic acid during pregnancy is associated with hypomethylation in the DNA sequence of *IGF2* DMR, a decrease that may differ by sex and race/ethnicity

Different nutrients are able to influence and modify these processes and therefore the epigenetic pattern, in the mother and the fetus during pregnancy, which is the most sensitive period to epigenetic changes that will persist in adult life (126). Micronutrients such as folic acid, choline, methionine, and vitamin B12 are involved in the folate pathway. The synthesis of methionine provides the methyl groups to DNA methyltransferases DNMTs1,3a, and 3b as well as Histone methyltransferases (HMTs), necessary for the global regulation of gene expression. Therefore, a deficit of one of these bioactive compounds alters the pattern of gene expression during development (127, 128). For this reason, the diet of pregnant women is always supplemented with folic acid (129). Moreover, antioxidants such as polyphenols, present in fruits like grapes and vegetables, green tea (EGCG), genistein, and curry, act by inhibiting DNMTs. These enzymes control the levels of oxidative stress, which negatively influences the metabolic profile during fetal development, predisposing the fetus to pathogenic alterations (130, 131).

Maternal malnutrition has been linked to epigenetic alterations such as the methylation (silencing) of the IGF2 gene, a key growth factor in fetal development (123). Higher levels of methylation in genes related to hepatic and cardiac function (CACNA1C and PDE1A related to intracellular calcium balance), inflammation (IL10, IL6), lipid/carbohydrate metabolism such as ABCA1 (related to cholesterol homeostasis), LEP (which regulates the synthesis of leptin that controls body weight), and GNASAS (which regulates hormonal metabolism) also appear associated with suboptimal maternal nutrition (132, 133). In accordance with Barker's hypothesis, other studies indicate that maternal malnutrition generates precocious insulin resistance which may accelerate early postnatal growth by shifting toward body fat instead of muscle mass (134). Some studies have associated DNA methylation in obesity markers with infant adiposity. Godfrey et al. (135) found a positive association between high methylation levels of RXRα and NOS3 genes in umbilical cord cells and adiposity in infants. On the other hand, a maternal high-fat diet present in BN and BED correlates to the origin of other metabolic diseases as hypertension and diabetes through changes in the hypothalamic region. A diet rich in fats also modifies the methylation status and the expression of genes related to the mesocorticolimbic reward circuit (dopamine and opioids) (136). Hyper-methylation of Leptin receptor, melanin-concentrating hormone receptor 1 (MCHR1), and proopiomelanocortin (POMC) was also related to an increase in body mass index (BMI) and a higher risk of obesity (137), also modifying energy homeostasis in the offspring (138). The permanent deregulation of the hypothalamic circuits promotes resistance to insulin and leptin, generating an increase in overweight status and food intake.

Other genes silenced by a high-fat diet and recently related to obesity are FYN (a member of the Src family of non-receptor tyrosine kinase related to inflammation, adipocyte differentiation, and insulin signaling), TAOK3 (an activator of the protein kinase MAPK cascade which affects fundamental cellular signaling pathways), PIWIL4 (a regulator of adipocyte proliferation) (139), and SIRT1 (expression stimulates the metabolism of fats in adipocytes by suppressing the Peroxisome proliferator-activated receptor PPARγ, also participating in the regulation of glucose homeostasis, anti-inflammatory activity and oxidative stress) (140). Therefore, a maternal diet rich in fat decreases the expression of SIRT1 in the fetal liver and heart, altering the fetal metabolism and generating a tendency to increase body fat.

Nutritional deficits associated with low protein intake typical of AN patients show harmful effects on fetal growth and a higher risk of obesity, diabetes, and hypertension in the offspring. These effects are caused by a deficit of folic acid, choline, and methionine. The deficiency of gestational proteins generates: hypomethylation of the *Wnt2* promoter in the placenta associated with fetal growth alterations (141); decreased expression of IGF-I and IGF-II producing hyperglycemia (142); hypermethylation of the PPARγ and Glucocorticoid receptor (GR) promoters involved in the regulation of blood pressure and in the metabolism of lipids in adults (143); increased expression of angiotensin II which generates an increase in blood pressure (142) and decreased expression of the agtr1b gene involved in hypertension.

A maternal protein-restricted diet during pregnancy is also associated with hypermethylation of key adipogenesis genes such as the peroxisomal proliferator-activated receptor-α (PPAR-α) related to cardiomyopathy in the offspring and liver X-receptor promoter (LXR)α involved in glucose homeostasis in the fetal liver (144). In addition, diets with protein deficits regulate the expression of microRNA related to chronic inflammation in offspring (145), as well as the over-expression of the transcription factor C/EBPs (CCAAT-enhancer-binding protein) which regulates the expression of genes involved in energy homeostasis (146).

In the gene-environment interactions, the extreme nutritional conditions associated with EDs such as AN, BN, and BED, have been deeply studied in the pregnancy period, because these disorders alter the epigenetic pattern of the mother and the developing fetus (147). Several studies postulated that environmental factors that favor thinness can lead to the overexpression of genes that suppress appetite and/or reduce body weight in individuals that are already genetically susceptible to low BMI and reduced percentages of body fat. When the complete genome methylation status is considered, instead of specific promoter regions of candidate genes, patients with anorexia and also patients with bulimia show a clear hypomethylation in their epigenome compared to healthy controls (118, 122). This phenomenon is produced by the deficit of folate and methionine, with a consequent decrease in the activity of DNMTs and HMTs, low protein intake, and very low levels of glucose and cholesterol in the blood (148). A recent study performed in 21 active ED, 43 past ED and 126 controls concluded that offspring of women with an active restrictive ED during pregnancy showed lower global methylation pattern in their genome compared to offspring of women with past restrictive ED. This study also demonstrated a decreased methylation at the DHCR24 locus in offspring of women with active ED during pregnancy and an increased methylation at the LGALS2 locus in offspring of women with past ED compared to controls (149).

Some differences in the expression of genes related to dopamine metabolism have been observed in patients with anorexia. An increase in the expression of the dopamine transporter SLC6A3 and a decrease by hypermethylation of the dopamine receptor D2 (DRD2) promoter have been associated with the reward effect in individual diagnosed with AN (119).

Along the same line, the hypermethylation of the POMC gene, a key regulator of appetite, and also the oxytocin receptor gene OXTR, negatively associated with BMI, has been observed in patients with AN (120). Moreover, a decrease in the mRNA levels of the glucocorticoid receptor gene NR3C1 has also been observed in the anorexic population (121). Finally, maternal psychosocial stress may alter placental imprinted gene expression, and lead to suboptimal fetal growth (150).

Other environmental factors such as smoking, alcohol, and drugs of abuse, which show higher percentages of consumption in ED patients than general population (151–153) may promote alterations in the epigenetic pattern of the mothers diagnosed with ED and their offspring (154). In a cohort of 40 patients diagnosed with EDs, Ehrlich et al. demonstrated than the methylation state of the Pro-opiomelanocortin (POMC) promoter was negatively affected by smoking but it was not influenced by the nutritional status (120). The co-occurrence of smoking and ED modify the epigenome during pregnancy affecting fetal development. A Recent study determined the effect of smoking during pregnancy in the hypomethylation of the Aryl hydrocarbon receptor repressor gene (AHRR) observed in neonates (155). Moreover, prenatal alcohol exposure produces severe impairments in fetal development which can result in fetal alcohol spectrum disorders (FASD). Alcohol can alters one carbon metabolism, critical for methylation of DNA, directly inhibiting the key enzymes methylenetetrahydrofolate reductase (MTHFR) and methionine synthase (MTR) (156, 157). Furthermore, recent studies have demonstrated in prenatal alcohol exposed children a differential methylation pattern in the genes SLC6A3 and DRD4 related to the dopaminergic system; in HLADPB1 and CD11A related to the immune response; and in H19 and SLC22A18 genes (158–160).

It is important to mention that some of the hormonal imbalances observed in EDs patients, as high cortisol and low sex hormones levels, can be promoted by environmental factors and they can influence the dynamics of the gene expression. These hormones can behave as direct ligands to promoters or key regulators which interact with transcription factors, altering the expression pattern of the epigenome (161, 162).

A large amount of epigenetic studies has focused on AN whereas the studies about BN are less abundant, and, in the case of BED, there are only few publications in human subjects. Additionally, most of these studies have small sample sizes, so it is necessary to perform studies on a larger scale to confirm these results. Finally, EDs are mainly brain disorders, so that some epigenetic alterations are specific of the nervous tissue. However, the majority of the published studies are performed using saliva or blood samples, which is a limitation to take into account for future studies.

### EDs During Pregnancy and Maternal Psychopathology (See Table 6)

Comorbid emotional disorders are common in women with EDs who typically experience depression, anxiety, or obsessive compulsive disorder (168). Recent evidence shows higher rates of depression and anxiety throughout pregnancy and during the postpartum period in women with active EDs related to their shape and weight concern (88, 89). Moreover, childbirth may trigger or relapse the onset of a comorbid psychiatric illness in the mother. Altered mood may impair the normal course of pregnancy and fetal development leading to adverse perinatal outcomes such as prematurity, SGA and IUGR babies (110). Furthermore, the post-partum period is the one at highest risk for worsening a depressive disturbance, particularly in women with an ED (84, 166), because infant feeding, maternal-child bonding, and the desire of weight loss, may exacerbate ED symptoms and lead to a mood disorder. Maternal adjustment during the first 3 months after delivery was clearly impaired and related to mental disturbances in mothers with EDs before pregnancy (164). Misuse of alcohol at conception, lower BMI, higher frequency of binging after delivery, and a history of AN, increased the risk of post-partum depression symptoms (169).

**Table 6:** Studies about comorbid psychopathology among pregnant women with eating disorders.

**Author (year)**	**References**	**Aim of study**	**Type of study. Population**	**Key results**
Franko et al. (2001)	(84)	To report pregnancy complications and neonatal outcomes for 49 live births in a group of women with eating disorders	Longitudinal *n* = 49	34.7% of women experienced postpartum depression
Mazzeo et al. (2006)	(163)	To investigate the associations among eating disorders, depressive symptoms during pregnancy and postpartum(PPD) and perfectionism in a population-based sample of women	Twin-based study *n* = 1,119	BN (OR 3.5, 95% confidence interval [95% CI]. 1.3–9.6) and BED (OR 2.8, 95% CI 1.1–7.0) diagnoses were strongly associated with PPD symptoms as assessed by the screening item. However, AN was not (OR 1.3, 95% CI 0.51–3.3).
Koubaa et al. (2008)	(164)	To study early adaptation to motherhood in mothers with EDs before pregnancy	Cross-sectional AN = 24 BN = 20 Controls = 67	92% of mothers with EDs before pregnancy reported problems regarding their maternal adjustment compared to 13% in the control group (*p* < 0.001), whereas there were no differences between the subgroups of ED and between those with and without verified relapse of ED during pregnancy. 50% of mothers with previous ED reported that they had been in contact with health services after delivery because of depression or other mental problems The exact odds ratios after adjustment for depression: 25 (95% CI 2.4-infinity) for AN of restrictive type, 31 (95% CI 5.4–341) for AN of binge eating type, 116 (95% CI 17-infinity) for BN (*p* < 0.01, *p* < 0.001, and *p* < 0.001, respectively)
Micali et al. (2011)	(165)	To investigate the effect of past depression, past and current eating disorders (EDs) on perinatal anxiety and depression in a large general population cohort of pregnant women	Longitudinal Cohort *n* = 10,887 Past-ED with (*n* = 123) and without past depression (*n* = 50), pregnancy ED symptoms with (*n* = 77) and without past depression (*n* = 159), past depression only (*n* = 818) and controls (*n* = 9,660).	Women with a history of EDs had increased levels of anxiety and depression during the antenatal and postnatal periods. This was most marked in the group with pregnancy ED symptoms and past depression (b coefficient:5.1 (95% CI: 4.1–6.1), pb 0.0001), especially at 8 months post-partum
Meltzer-Brody et al. (2011)	(166)	To examine the prevalence of comorbid eating disorders (EDs) and trauma history in women with perinatal depression. Patient Health Questionnaire (PHQ-9), Edinburgh Postnatal Depression Scale (EPDS),	Cross-sectional and correlational *n* = 158	Women with BN reported more severe depression during perinatal period. BN: EPDS score, 19.1, standard deviation [SD 4.3], *p* = 0.02; PHQ-severity 14.5, SD 7.4, *p* = 0.02 Controls: EPDS score 13.3, SD = 6.1; PHQ 9.0, SD = 6.2)
Easter et al. (2015)	(167)	To investigate longitudinal patterns of psychopathology during the antenatal and postnatal periods among women with current and past ED	Observational prospective Current ED (C-ED) *n* = 31 Past ED (P-ED) *n* = 29 Controls *n* = 57	- Women with a C-ED at the start of pregnancy showed an overall pattern for decreasing levels of psychopathology among the antenatal and postnatal periods. - Symptoms of ED: C-ED (b coeff: 1.3,95% CI: 1.1–1.5) and P-ED groups (b coeff: 0.6, 95% CI: 0.4–0.8) had higher total EDE-Q scores compared with healthy controls - Anxiety: C-ED and P-ED had higher state (C-ED = b coeff: 0.5, 95% CI: 0.4–0.7; P-ED = b coeff: 0.3, 95% CI:0.1–0.4) and trait (C-ED = b coeff: 0.5, 95% CI: 0.4–0.6; P-ED = b coeff: 0.2, 95% CI: 0.1–0.4) anxiety scores, compared with healthy controls - Depression C-ED (b coeff: 2.0, 95% CI: 1.5–2.6) and P-ED (b coeff: 0.9; 95% CI: 0.5–1.4) had higher depression scores at all time points than control group

Easter et al. (167) investigated longitudinal patterns of psychopathology during the antenatal and postnatal periods among women with current and past EDs. They found decreasing levels of psychopathology at the start of pregnancy in women with current ED. However, symptoms of anxiety, and depression remained high at all pregnancy stages. In contrast women with a past ED were at risk of increasing psychopathology throughout pregnancy and during the postnatal period. The strengths of this data were the longitudinal design and the strict inclusion criteria of EDs, but the main limitation was the small sample with insufficient power to detect potential differences. Mazzeo et al. (163) assessed the association among EDs, depressive symptoms, and perfectionism during pregnancy or post-partum in a population-based sample of pregnant women. This data showed a higher rate of depressive symptoms during pregnancy and post-partum in women with a history of EDs, in particular in women with BN and BED, and the authors found a relationship between symptoms of depression and aspects of perfectionism. One limitation of this study was the lack of information regarding the timing of the beginning of the depressive symptoms and the diagnostic criteria of post-partum depression. Along the same line, Micali et al. (165) investigated the effect of past depression, and past or current ED, on perinatal anxiety and depression in a large general population cohort of pregnant women. They found that women with a history of an ED had increased levels of anxiety and depression during the antenatal and postnatal periods. ED symptoms and past depressive episodes were related to the highest risk for a depressive and anxiety disorder during pregnancy. Data was limited to a self-reported assessment of psychiatric history. One possible explanation of these results is that women with EDs are predisposed to develop an affective disorder and this condition together with the stressful stage of pregnancy, aggravated by body image concern, weight gain, and loss of control, may trigger a psychiatric illness (28). Another possible reason may be associated with nutritional deficiencies, for example zinc deficiency has been linked to depression symptoms (58). Low levels of micronutrients like folate, vitamin D, vitamin B12, iron, selenium, and fatty acids, which are essential for the biosynthesis of several neurotransmitters (serotonin, dopamine, and norepinephrine) have been linked to perinatal depression (170). Furthermore, elevated levels of cortisol described in patients with EDs have been associated with most forms of depression (171).

### The Effects of EDs on Postpartum Course, Breastfeeding, and the Composition of Human Milk

Kouba et al. (164) described that women with EDs had more problems regarding their adjustment to the new maternal situation at 3 months post-partum. Although the prevalence of postpartum remission was high, EDs continued in a large proportion of women and, in the postpartum period, women with EDs experienced a decrease in BMI during the first 6 months after delivery (65).

If the onset of EDs was before pregnancy the proportion remitting was significantly low. The presence of binge symptoms or compensatory behaviors during pregnancy, expressed a more severe form of BN, which persisted in the postpartum period. Higher BMI and psychological dysfunction were associated with continuation of BED during postpartum (172). Furthermore, a retrospective case-control study demonstrated that a third of women with EDs developed postnatal depression increasing the risk for relapse of EDs (88).

Some studies showed an increased rate of breastfeeding among women with current or prior EDs related to the motivation and the desire for weight loss, while other studies found low incidence (20, 165). Nevertheless, mothers with active or paste EDs reported problems when giving human milk, such as scarce lactation and precocious finalization of breastfeeding (46). Torgersen et al. (173) conducted a large population-based study and reported that women with EDs started breastfeeding in the same proportion as women without EDs. However, women with EDs were more likely to stop breastfeeding earlier than the control group. Women with BED had a lower rate of lactation. A possible explanation was that women with EDs felt embarrassment when breastfeeding and they showed high shape dissatisfaction and lower self-esteem. Moreover, they had low social support and felt depressed when breastfeeding. Stress has also been related to the short duration of lactating period (174). Low leptin levels in ED patients could influence the capacity to regulate the mechanisms of satiety in breastfed infants, leading to lactation failure (175). Squires et al. (176) showed that ED women were likely to have less mother-infant interaction during feeding. They described that such mothers were less sensitive when feeding their babies, more stressed, and with dysregulated interaction patterns.

To our knowledge there is a lack of studies regarding the composition of human milk in ED patients. A recent study described that the variance in milk fat content has been associated with BMI, and maternal body composition may be related to the nutritional value of human milk (177). We may speculate that mothers with an ED have low pre-pregnancy nutritional status that can lead to low maternal fat stores for lactation, and low nutritional amounts for the baby. For example, lower iron and vitamin B12 intake in mothers with an ED may cause deficiency of these elements in breastfed infants with consequent neurological disabilities (178). Moreover, low maternal vitamin D status described in AN patients may affect vitamin D quantities received by the breastfed neonate (179).

### EDs During Pregnancy: Risk Factors and Psychological Intervention

The most important risk factors linked to the onset of EDs during pregnancy are: (24, 102) personality characterized by low self-esteem and perfectionism; mood disorders such as depression, anxiety and obsessive-compulsive disorder; a history of social dysfunction, maltreatment or sexual abuse; toxic consumption like smoking, drugs of abuse and alcohol; inadequate or excessive stres; greater BMI changes from pre-pregnancy period; unplanned pregnancy and lower marital and social support. The major risk factor is a past history of EDs or family history of EDs (180).

Psychological intervention focused on symptoms of dietary restriction and overevaluation of shape and weight, helps the patients to establish a pattern of regular eating, avoid compensatory behaviors and reducing dieting in order to address motivation (181).

Nevertheless, there is a lack of clinical trials about psychological intervention in pregnant women with EDs. The treatment includes Cognitive Behavioral Therapy (CBT) and Interpersonal Psychotherapy (IPT) which have been used as treatments for prenatal and postnatal depression demonstrating potential benefits but it is difficult to know which is the most effective (14, 182). The goal is to maintain regular eating patterns and optimize nutritional intake for the mother and fetus (183) The body of evidence supporting IPT use is far more modest than it is for CBT (184). CBT reached higher rates of remission in patients with BN and BED (185).

### EDs During Pregnancy: Target Therapy

Based on current evidence pregnancy is a critical period for an intervention in EDs women which should be strictly monitored prior, during, and after pregnancy. Nonetheless, there are several challenges for clinicians. The best approach for pregnant women with EDs implies a multidisciplinary management comprised of gynecologists, midwives, nutritionists, and mental health professionals working as a team. However, there is an insufficient medical training to openly discuss eating behaviors with the patient (29). Women with EDs do not often disclose this information to clinicians due to fear of stigmatization or misunderstanding. Some questionnaires are applied to ED identification, but none of them are specific to pregnant population. UK guidelines (NICE) suggest asking one or two specific questions. The SCOFF questionnaire has been also proposed (6). Recently, Emery et al. reported a structured clinical interview to assess disordered eating patterns among overweight and obese pregnant women [The Eating Disorder Examination Pregnancy Version (EDE-PV)] (186). Identifying women with EDs but with normal weight is still more difficult.

Otherwise pregnant women with EDs show a specific nutritional patterns: many of them are vegetarian (or with a lower intake of meat), they smoke and consume high amounts of coffee and do not show significant deficiencies in mineral and vitamins (60). Moreover, dietary supplements are similar between pregnant women with and without EDs (187). Therefore, pregnant women with EDs should receive accurate information from a dietitian about a balanced diet involving food from each nutritional group correctly represented and also about daily energy requirements, not only during pregnancy, but also during post-partum and breastfeeding (80).

It is also important to mention that the use of psychotropic, analgesic, and gastrointestinal medication should be assessed (188). AN or recurrent self-induced purging in the absence of binge eating (EDNOS-P) was directly associated with the use of anxiolytics and sedatives post-partum (adjusted RR: 5.11, 99% CI: 1.53–17.01 and adjusted RR: 6.77, 99% CI: 1.41–32.53, respectively). The use of analgesics was higher in pregnant women with BED, and laxatives were also used by all women with EDs, before, during, and after pregnancy. In severe cases of BN, antidepressant medication could be needed. The most common are selective serotonin reuptake inhibitors (SSRIs) (14). Clinicians should be encouraged to query about patients' medication and provide evidence-based counseling about the risks of treatment exposure vs. the risks of untreated psychiatric diseases.

## Discussion (Table 7)

The incidence of EDs has increased globally in the last years, particularly in developed countries. These disturbances start at an early age in adolescent girls due to the social pressure about body shape stereotypes and the impact of beauty canons showed in social programs or mass media. Slimness reflects healthy nutritional habits if it is not associated with self-destructive behaviors, emotional dysregulation, excessive pursuit of thinness, and restrictive and bulimic eating patterns, typical of EDs. The combination of body changes during puberty, anxiety, and psychological disorders may trigger the first episodes of vomiting and purging, becoming into pathologically dysfunctional eating behaviors (55).

**Table 7:** EDs during pregnancy: bullets points.

Periconceptional period	• High rate of unplanned pregnancy • Infertility • Hypotalamic and pituitary dysfunction • PCOS (BN)
Preconception Nutritional profile	• Micronutrients and vitamin deficiencies • Anemia, leukopenia, trombocytopenia • Low mineral density
Nutritional profile during pregnancy	• Similar dietary patterns to general population • High diet quality score in ED population (more vegetarian) • BED: greater intake of total energy and fat • Higher amount of caffeine • Greater risk for gestational anemia • Higher risk of micronutrient deficiencies
GWG	• GWG depends on the relapse of ED symptoms during pregnancy • Lower GWG in hospitalized patients if compared to general population
Endocrine manifestations	• Hypotalamic and pituitary dysfunction • Suppressed thyroid function • Low leptin and peptide YY levels, high ghrelin levels • Increase insulin sensitivity, high adiponectin levels • High cortisol and CRH levels
Pregnancy outcomes	Increased risk of: • Spontaneous abortion and preterm delivery • BED y BN: greater rate of C-section
Fetal outcomes	Increased risk of: • IUGR • SGA • LGA (BED subtype) • Perinatal mortality (few studies) • Microcephaly and intraventricular hemorrhage (few studies)
Maternal psychopatology	• Depression • Anxiety • Post-partum depression
Postpartum period	• Depression • Stress • Earlier stop of breatsfeeding

Stress and anxiety may play a critical role in the onset of EDs. Binges and purging episodes are described more likely to happen in response to environmental stressors. Stress promotes long-term maladaptive modifications in neural circuits that regulate food intake, included neuroendocrine and sympathetic pathways (189).

Patients present a deficit in emotion regulation abilities in response to an acute psychosocial stressor (190). Stress itself may complicate the course of pregnancy and can cause preterm deliveries or intrauterine growth restriction, regardless the diagnosis of ED (191).

Women with EDs may display severe malnutrition, leading to hypothalamic dysfunction, low bone mass and precocious osteopenia (44, 62). Moreover, patients with AN have been found to have at least one vitamin and trace element deficiencies suggesting an alteration of micronutrient status (71).

In this line diet is one of the most important factors during pregnancy, which may program the health of the offspring. Therefore, unbalanced diet may predispose the developing fetus to several diseases in adulthood as the demand of macro and micronutrients increases during pregnancy (192–195). Furthermore, a malnutrition status as well as alterations in hormonal pathways may reduce but no abolish fertility in ED women, leading to an insufficient use of contraception and therefore producing a high incidence of unplanned pregnancy. This condition promotes an additional risk of impaired fetal development due to the lack of concern about prenatal care (abstinence from alcohol, no smoking, multivitamin supplements, and balanced nutrition) (42).

Pregnancy represents a period of increased vulnerability, which may trigger dysfunctions on the eating patterns or may be a chance to improve and treat EDs due to the concern for the unborn baby and a different perception of body image during gestation.

Our systematic review displays a global overview about the impact of EDs during pregnancy explaining its multifactorial mechanisms.

Patients with EDs show adverse pregnancy outcomes such as miscarriage, preterm delivery, or fetal disabilities as poor fetal growth or malformations. However, the multifactorial origin of EDs as well as the limited experimental studies performed in humans make difficult to establish a clear relationship between EDs during pregnancy and birth impairments. Maternal malnutrition and poor GWG suggesting a relapse of EDs symptoms alter the fetal supply of nutrients and trigger the deregulation of several hormonal pathways, increasing the risk of damage to intrauterine environment. An altered nutritional pattern in mothers, also induce epigenetic changes in the global expression pattern of the fetus, which will trigger biological and psychological alterations in offspring lifelong outcomes. Additionally, substances of abuse such as alcohol, narcotics, or tobacco, more frequent in EDs patients, may have a comorbid impact on fetal development. These environmental factors also produce changes in the global pattern of gene expression as well as hormonal imbalances. So that, they represent some of the multifactorial variables that strongly influence the epigenetic profile of the mothers with EDs and their offspring.

Considering the evidence that previous and most severe EDs are at the highest risk of fetal impairment, it is imperative to detect altered eating patterns in childbearing age women during the pre-conception period. Once diagnosed, a multidisciplinary team have to start the treatment for the specific ED detected, which includes nutritional support, a screening related to alcohol and drugs of abuse, psychotherapeutic techniques, and the use of psychotropics if necessary. Early detection is essential to prevent malnutrition, mental and somatic comorbidities, and the relapse of symptoms during gestation with consequent poor obstetric and fetal outcomes. An early intervention will empower the woman to her own care and would avoid lifelong morbidity for both mother and fetus.

The present study has been performed following the PRISMA methodology for systematic reviews and the authors' clinical experience.

However, some of the studies explored in this review show opposite results probably because they investigated different populations with different health conditions: for example, hospitalized patients with severe EDs vs. a sample of general populations with self-reported EDs.

An important bias in the majority of the papers evaluated was the diagnosis of maternal EDs by self-reported screening and the dietary intake through a food frequency questionnaire. The main limitations of these questionnaires were the different food lists and the possible mistakes in self-reporting. Moreover, the self-reporting method for determining the inclusion criteria of EDs usually underestimate their prevalence as well as some important features such as mental mood, GWG, and nutritional status of the mother. Otherwise the fetal adverse outcomes have multifactorial causes, so a combination of several comorbid disorders may impair fetal development such as undernutrition, psychiatric pathologies, or substance abuse besides EDs. Moreover, a large number of studies did not distinguish between past or active EDs and several studies did not adjust for confounding factors such as smoking, drugs, pre-conception maternal BMI or the severity of the illness, generating biases.

Pregnant ED patients may display a good gestational evolution if they reach a healthier BMI preceding pregnancy, a correct GWG, a balanced nutrition, and a healthy mental state of the mother.

Nevertheless, the recognition of dysregulated eating patterns is a challenge for clinicians due to the lack of a standardized screening method during pregnancy. EDs impair gastrointestinal function leading to esophageal acidic damage for self- inducing vomiting, gastric distension, dysphagia, increased intestinal motility and chronic diarrhea. Clinician should detect such symptoms and practice the differential diagnosis with intestinal disease like celiac disease, gastroesophageal reflux, and inflammatory bowel diseases (196).

Clinicians have to be alert to recognize the psychological features associated with the onset of EDs during gestation, reinforcing the positive changes of body image in pregnant women and focusing on self-nurturing interventions to reduce stress and anxiety (15). Clinicians have also to motivate pregnant women to supplement essential nutrients such as iron, folate, and iodine for an adequate fetal development.

This target intervention must be continued in the postpartum period, which is the most vulnerable stage for the relapse of EDs symptoms and for the overlap with psychiatric disorders. Mothers with EDs also experience stress during the breastfeeding period, therefore a strict postpartum follow-up is necessary.

Finally, it is also necessary to clarify the biological mechanisms that promote harmful effects on the fetus of mothers diagnosed of EDs. Further prospective studies with large sample sizes are needed, discriminating the consequences of the different subtypes of EDs. Human trials would also determine appropriate approaches to treat these disorders in order to design structured interventions during gestation.

## Author Contributions

GS conceptualized, designed, and drafted the initial manuscript and tables supported by the rest of the authors. VA-F supervised the methodology. GS and VA-F reviewed the manuscript. VA-F, VA-B, AH, XM, EM, MA-V, AB, and SF-M contributed to bibliographic sources as well as the elaboration of the initial manuscript and tables, performing the different topics explored in this review. GS, MG-R, OG-A, and VA-F conceptualized and coordinated the review. MG-R and OG-A performed the final check of the manuscript, respectively, from gynecologist's and neonatologist's point of view. All authors critically reviewed the manuscript and approved the final manuscript as submitted.

## Conflict of Interest

The authors declare that the research was conducted in the absence of any commercial or financial relationships that could be construed as a potential conflict of interest.

## Supplementary Material

Table S1 Dietary Reference Intakes (DRIs) during pregnancy recommended by the Institute of Medicine (IOM).
