# Supplementary material for: Eating Disorders During Gestation: Implications for Mother's Health, Fetal Outcomes, and Epigenetic Changes
Source: Front Pediatr. 2020 Sep 17;8:587. doi: 10.3389/fped.2020.00587 (PMC7527592; doi:10.3389/fped.2020.00587)
Supplement: Table S1 — Dietary Reference Intakes (DRIs) during pregnancy recommended by the Institute of Medicine (IOM). [file Table_1.docx]

|  |  | |  |  |  |
| --- | --- | --- | --- | --- | --- |
|  | **Pre-pregnancy** | **Pregnancy** |  |  |  |
| **Nutrient** | **RCOG/IOM** | **RDA american** | **DRI/IOM** | **EFSA** | **FIGO** |
| Energy (kcal/d) | 1940 | 2099 +340, since 2^nd^ trim. | +340 2^rd^ trim, +452 3^rd^ trim | + 70 1^st^ trim; +260 2^nd^ trim; +500 3^rd^ trim. | + 69 1^st^ trim;+266 2^nd^ trim; + 496 3^rd^ trim. |
| Protein (g/d) | 45 (0.8g/kg/d) | 71(1.2g/kg/day) | 60 (1.1g/kg/d) | + 26 | 71 |
| Carbohydrate (g/d) | 130 | 175 | 175 | 175 | 175 |
| Thiamin (mg/d) | 0.8/1.1 | 1.4 | 1.4 | 0.8 | a |
| Riboflavin (mg/d) | 1.1 | 1.4 | 1.4 | 1.5 | a |
| Vitamin C (mg/d) | 40/ 75 | 85 | 85 | 85 | 105 |
| Vitamin E (mg/d) | 15 | 15 | 15 | 11 | 15 |
| Folate (µg/d) | 200/ 400 | 600 | 600-800 | 600 | 600 |
| Vitamin D (µg/d) | 5 | 15 | + 10 | 15 | 15 |
| Vitamin A (µg/d) | 600/700 | 770 | 770 | 700 | 750-770 |
| Niacin (mg/d) | 13/ 14 | 18 | 18 | 16 | a |
| Vitamin B6 (g/d) | 1.2/ 1.3 | 1.9 | 1.9 | 1.5 | 1.9 |
| Vitamin B12 (µg/d) | 2.4 | 2.6 | 2.6 | 4.5 | 2.6 |
| Vitamin K (mg/d) | 90 | - | 90 | 70 | - |
| Calcium (mg/d) | 700/ 1000 | 1000 | 1000 | 1000 | 1000-1300 |
| Magnesium (mg/d) | 270 | 350 | a | 300 | a |
| Sodium (mg/d) | 1600 | 1500 | a | a | a |
| Chloride (mg/d) | 2500 | 2500 | a | a | a |
| Potassium (mg/d) | 3500 | 4700 | a | 3500 | a |
| Iron (mg/d) | 15/ 18 | 27 | 27 | 27-30 | 27 |
| Zinc (mg/d) | 7/ 8 | 11 | 11 | + 1.6 | 11-12 |
| Copper (mg/d) | 1.2 | 1 | 1 | 1.5 | 1 |
| Selenium (µg/d) | 60 / 55 | 60 | 60 | 70 | 60 |
| Iodine (µg/d) | 140 | 200 | 200 | 200 | 220 |
| DHA(mg/d)/ Ω3(g) | 250/ 2 | + 200 / 1.4 |  | +100-200 | +200/ 1.4 |
| Phosphorus (mg/d)  a= no increment during pregnancy | 550/ 700 | 700 | 700 | 550 | 550 |

**Supplemental Table 1 (S1).** Dietary Reference Intakes (DRIs) during pregnancy recommended by the Institute of Medicine (IOM).^192,194^ ^195,196^.
